# Nonoptimal Gene Expression Creates Latent Potential for Antibiotic Resistance

**DOI:** 10.1093/molbev/msy163

**Published:** 2018-08-28

**Authors:** Adam C Palmer, Remy Chait, Roy Kishony

**Affiliations:** 1 Department of Systems Biology, Harvard Medical School, Boston, MA; 2 Laboratory of Systems Pharmacology, Harvard Medical School, Boston, MA; 3 Institute of Science and Technology Austria, Klosterneuburg, Austria; 4 Departments of Biology and Computer Science, Technion—Israel Institute of Technology, Haifa, Israel

**Keywords:** antibiotic resistance, evolution, gene expression, systems biology

## Abstract

Bacteria regulate genes to survive antibiotic stress, but regulation can be far from perfect. When regulation is not optimal, mutations that change gene expression can contribute to antibiotic resistance. It is not systematically understood to what extent natural gene regulation is or is not optimal for distinct antibiotics, and how changes in expression of specific genes quantitatively affect antibiotic resistance. Here we discover a simple quantitative relation between fitness, gene expression, and antibiotic potency, which rationalizes our observation that a multitude of genes and even innate antibiotic defense mechanisms have expression that is critically nonoptimal under antibiotic treatment. First, we developed a pooled-strain drug-diffusion assay and screened *Escherichia coli* overexpression and knockout libraries, finding that resistance to a range of 31 antibiotics could result from changing expression of a large and functionally diverse set of genes, in a primarily but not exclusively drug-specific manner. Second, by synthetically controlling the expression of single-drug and multidrug resistance genes, we observed that their fitness–expression functions changed dramatically under antibiotic treatment in accordance with a log-sensitivity relation. Thus, because many genes are nonoptimally expressed under antibiotic treatment, many regulatory mutations can contribute to resistance by altering expression and by activating latent defenses.

## Introduction

Regulation of gene expression contributes to the ability of bacteria to survive antibiotics and other stresses (Miller and Sulavik 1996; Grkovic et al. 2002; Depardieu et al. 2007; Gooderham and Hancock 2009; Lister et al. 2009; Storz and Hengge 2010; Poole 2012). However, regulation is rarely perfect (Price et al. 2013); some genes important for survival may not be regulated by a given stress and even if they are regulated their stress-induced expression levels may not be best tuned to maximize growth and survival under the stress (Bollenbach et al. 2009). The defining feature of nonoptimality is the potential for improvement; a gene is nonoptimally expressed under stress if mutations that change its expression level can improve the ability to survive the stress. Thus, mutations that confer antibiotic resistance by changing or disrupting regulation can be understood as instances where the natural gene expression level, though possibly optimal in natural environments, is nonoptimal under antibiotic treatment.

Many cases are known where resistance results from mutations that change gene expression, but the extent of this phenomenon has not been systematically characterized. Mutations that confer resistance to antibiotics by altering the expression of either native or horizontally transferred resistance genes are common in clinical isolates and in laboratory evolution experiments (McCalla et al. 1978; Bergstrom and Normark 1979; Curtis et al. 1985; Flensburg and Skold 1987; Maneewannakul and Levy 1996; Koutsolioutsou et al. 2005; Depardieu et al. 2007). These genes have included those with specific functions in antibiotic resistance, such as drug degrading enzymes (e.g., beta-lactamases) and transcriptional regulators of stress response (e.g., *marA* and *soxS*), and also those not thought of as “resistance genes” per se but genes whose function is intrinsic to a drug’s mechanism of action, including drug targets themselves (e.g., *DHFR*, known in *Escherichia coli* as *folA*), enzymes that activate a prodrug (e.g., *nfsA*), and porins that mediate cellular entry of certain antibiotic molecules (e.g., *ompF*). Nonoptimal regulation can also be identified in chemical-genetic screens where gene deletion or overexpression libraries are tested for resistance to different antibiotics. Several large-scale screens of gene deletion libraries have been applied to stresses at subinhibitory intensities to identify hypersensitivity, rather than resistance, to identify a cell’s “intrinsic resistome” (Tamae et al. 2008), and to understand drug mode of action and gene function (Hillenmeyer et al. 2008; Girgis et al. 2009; Liu et al. 2010; Nichols et al. 2011; Lee et al. 2014; French et al. 2016; Shiver et al. 2016). However, any given gene in the intrinsic resistome may or may not have the capacity to contribute to the evolution of stronger levels of antibiotic resistance, which means that genetic screens for hypersensitivity (with drug concentrations below the wild type’s minimum inhibitory concentration, MIC) do not provide the same information as screens for resistance (testing drug concentrations above the MIC). Screens above the MIC have been applied to gene overexpression mutants and have typically applied a stringent selection (far above the MIC) to identify only the few most highly resistant mutants, often in search of drug target genes (Li et al. 2004; Pathania et al. 2009; Soo et al. 2011; Farha and Brown 2016). Because previous array-based methods are labor intensive (Breidenstein et al. 2008; Schurek et al. 2008; Fernandez et al. 2013), and the results of such screens depend on drug concentration with an informative concentration range that is highly variable between different drugs (French et al. 2016), we have been lacking an efficient and sensitive screen for nonoptimally expressed genes under antibiotic treatment.

Central to understanding the optimality of expression is how protein levels affect fitness, which is understood to be through a sum of benefits and costs of expression (Dekel and Alon 2005). Experimental evolution has shown that expression levels can rapidly evolve to the optimal solution of the fitness–expression function in a given environment, and thus this framework can produce predictive relationships between environments and optimal gene expression levels (Dekel and Alon 2005).

Therefore, to understand how hidden potential for antibiotic resistance is unrealized by a cell’s native regulatory program, we sought to investigate both how widespread is nonoptimal gene expression across a range of antibiotic mechanisms and how antibiotics change fitness–expression functions.

## Results

### A Rapid Genome-Wide Screen for Nonoptimal Gene Expression under Antibiotic Treatment

**Fig. 1.**
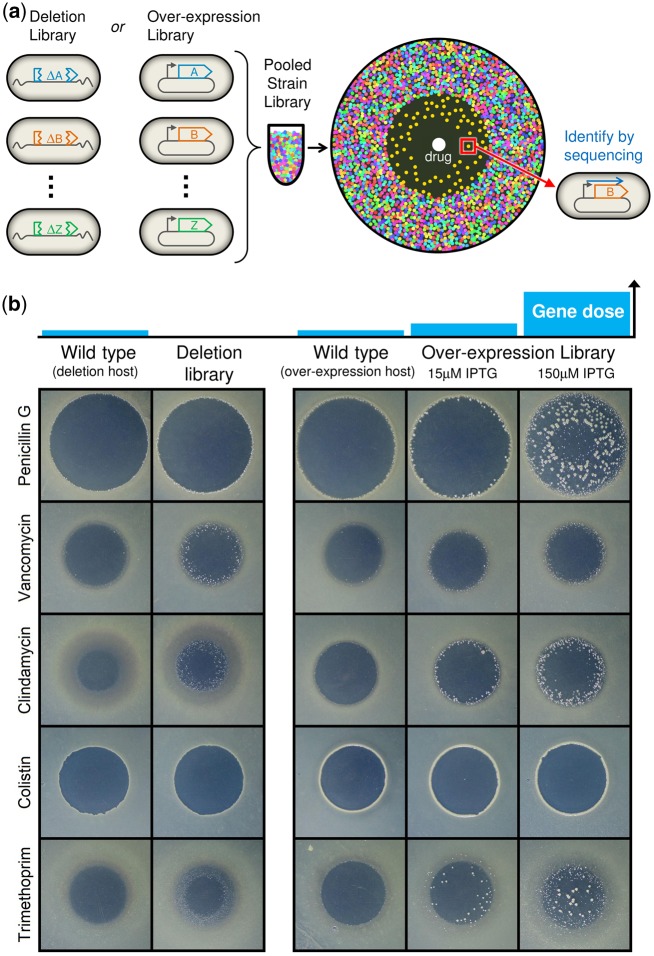
A genome-wide screen identifies changes in gene expression that confer antibiotic resistance. (*a*) A library of *Escherichia coli* strains with genes deleted or overexpressed is pooled and plated as a lawn on agar. A drug spot is applied which creates a zone of growth inhibition. Members of the strain library with increased drug resistance grow inside the zone of inhibition (yellow colonies), and are picked and identified by DNA sequencing. (*b*) Photographs of assay plates for five example antibiotics (out of 31) illustrate that both gene deletion and overexpression can confer drug resistance, and the possible levels of resistance range from none at all (e.g., colistin), to modest (e.g., clindamycin, vancomycin), to very strong (e.g., penicillin, trimethoprim). Plate images for all antibiotics are shown in supplementary figure S1, Supplementary Material online.

We developed a robust, rapid, and inexpensive genome-wide screen to identify nonoptimally expressed genes, that is, genes where a change in expression confers drug resistance. To identify both negative and positive expression changes that confer resistance, we screened two *E. coli* strain libraries: A gene deletion library (in which each nonessential gene is individually replaced by a Kanamycin resistance cassette) (Baba et al. 2006) and a gene overexpression library (a collection of plasmid-encoded isopropyl β-d-1-thiogalactopyranoside [IPTG]-inducible genes) (Kitagawa et al. 2005). The overexpression library was first transformed in pools into a wild-type MG1655 *ΔlacZYA*, conferring the favorable feature that each expression mutant is represented in the screen by the descendants of hundreds of unique transformation events. The libraries were screened on an antibiotic diffusion gradient, avoiding the need to fine tune the concentration of each drug and providing the sensitivity to detect both strongly and mildly resistant mutants. The assay comprises two steps (fig. 1*a*): 1) A strain library (deletion or overexpression) is pooled and seeded as a lawn onto a nutrient agar plate. An aliquot of concentrated antibiotic solution is spotted in the center of the plate, as in a classical disk-diffusion assay, and diffuses through the media forming a gradient of antibiotic concentrations. Following incubation, all strains with the wild-type resistance level grow into a dense lawn across the plate, except in a zone of clearing around the antibiotic spot, where drug concentrations are high enough to preclude visible growth. However, strains with enhanced resistance to the antibiotic grow at higher drug concentrations, closer to the drug spot, and thus appear as visible individual colonies inside the zone of inhibition. By selecting on solid agar the population of survivors is spatially separated, preventing the most resistant mutants from outcompeting slower-growing mutants of interest. 2) Drug-resistant colonies are picked and identified by Sanger sequencing of the expression plasmid or of the chromosome adjacent to the site of gene deletion. When the deletion or overexpression of a gene improves drug resistance, the gene is identified as having nonoptimally high or nonoptimally low expression, respectively, under antibiotic treatment.

We employed our assay on 31 antibiotics, representing most classes of antibiotics used in the treatment of Gram-negative bacteria (table 1), and observed many gene expression mutants providing resistance. For each of three expression conditions per antibiotic (deletion; mild overexpression, 15 µM IPTG; strong overexpression, 150 µM IPTG), a pooled diffusion screen was performed and 48 colonies were picked from inside the wild-type zone of inhibition and sequenced. The presence and abundance of drug-resistant gene expression mutants is highly variable across antibiotics, with very strongly resistant mutants appearing on some drugs (e.g., penicillin G and trimethoprim), while other antibiotics permitted no resistant expression mutants at all (e.g., colistin) (fig. 1*b*, supplementary fig. S1, Supplementary Material online). In contrast, plates inoculated with clonal wild-type cultures rarely show colonies in the zone of inhibition, representing infrequent spontaneous drug resistance mutations. The quantitative level of resistance, as measured by how far into the zone of inhibition colonies extended, was also highly variable across antibiotics, with gene overexpression commonly but not always capable of producing stronger resistance than gene deletion (supplementary fig. S2, Supplementary Material online). Colonies that grew the very farthest into zones of inhibition were cases of known, potent resistance determinants that have direct physical interactions with drugs or regulate directly interacting genes (*folA*, *ampC*, *marA*, and *soxS*). Over half of the 93 assays (31 drugs × 3 expression conditions) produced resistant colonies, for a total of approximately 2,400 colonies picked and sequenced. To avoid false identifications from the occurrence of spontaneous resistance mutations, we called gene–drug interactions only for genes that were detected in at least two separate colonies in the zone of clearing of the same antibiotic (average false discovery rate <1%, see supplementary note, Supplementary Material online). The robustness of the primary screen was tested for all genes identified in three drugs of different mode of action (clindamycin, sulfamethoxazole, and phleomycin), which together interacted with 28% of all hits. The drug diffusion screen was repeated for each hit using wild-type cultures inoculated with a 1:500 fraction of the mutant strain, where four drug-resistant colonies per assay plate were picked and tested for presence of the mutation (see Materials and Methods). This secondary validation confirmed the resistant phenotype for 34 of 37 genes tested, most often with 3/4 or 4/4 colonies picked being the inoculated mutant, conferring extremely high statistical significance (*P* < 10^−7^ given the initial mutant: wild-type ratio of 1:500) (supplementary table S2, Supplementary Material online).

**Table 1.** List of Antibiotics Used in This Study, Mechanism of Action, and Abbreviation.

Drug Class	Drug Name	Abbreviation
Cell wall synthesis		
Cephalosporin	Cephalexin	CLX
	Cefoxitin	FOX
	Cefsulodin	CFS
Glycopeptide	Vancomycin	VAN
Penicillin	Ampicillin	AMP
	Carbenicillin	CRB
	Mecillinam	MEC
	Penicillin G	PEN
Cell membrane		
Polypeptide	Colistin	COL
	Polymyxin B	PMB
Fatty acid synthesis inhibitor	Triclosan	TCL
Transcription		
Rifamycin	Rifamycin SV	RIF
Translation		
Aminoglycoside	Amikacin	AMK
	Streptomycin	STR
	Tobramycin	TOB
Macrolide	Azithromycin	AZI
	Erythromycin	ERY
	Spectinomycin	SPX
	Spiramycin	SPR
Lincosamide	Clindamycin	CLI
Tetracycline	Doxycycline	DOX
	Tetracycline	TET
DNA synthesis		
Quinolone	Ciprofloxacin	CPR
	Lomefloxacin	LOM
	Nalidixic acid	NAL
Folate synthesis inhibitor	Trimethoprim	TMP
	Sulfacetamide	SCM
	Sulfamethoxazole	SMX
Free radical production		
Glycopeptide	Bleomycin	BLM
	Phleomycin	PHM
Nitrofuran	Nitrofurantoin	NIT

### Novel and Functionally Diverse Pathways to Drug Resistance

**Fig. 2.**
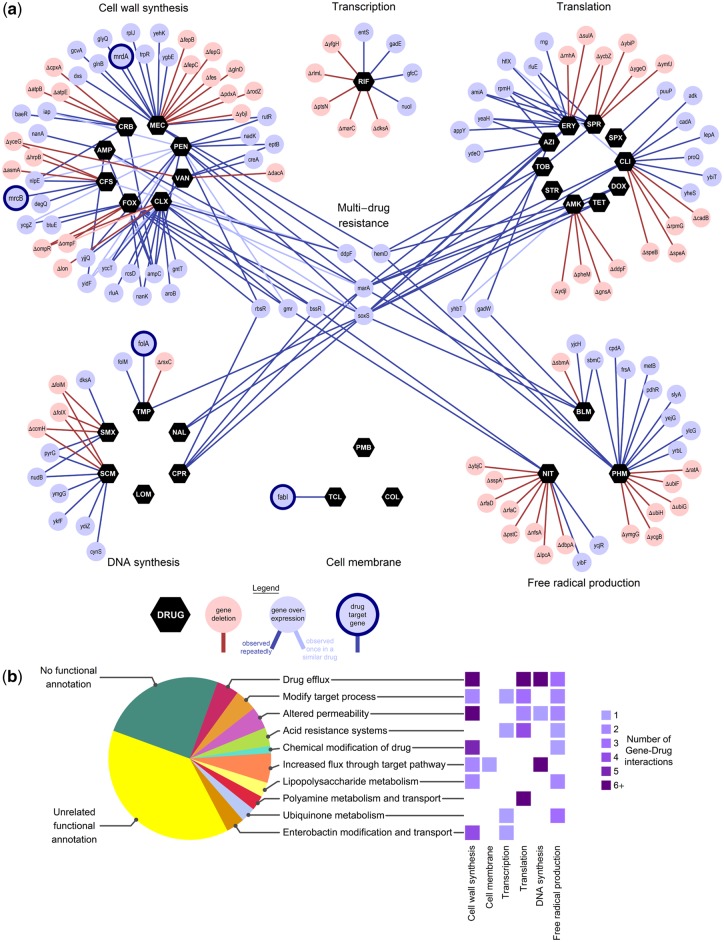
Drug-specific and drug-general resistance through a multitude of gene expression changes. (*a*) Antibiotics (black hexagons) are grouped by mechanism of action (see table 1 for abbreviations). *Escherichia coli* genes are marked by red circles when deletion confers antibiotic resistance and blue circles when overexpression confers antibiotic resistance; known antibiotic targets whose overexpression confers resistance are outlined in dark blue. Changes in gene expression that resist only one mechanism of drug action are grouped around the antibiotics of that mechanism, while those that resist multiple classes of drug are shown in the center. Pale-colored links denote changes in gene expression that were identified only once as resisting a particular drug, that are included because they were repeatedly observed to resist another drug of the same mechanism of action (supplementary note, Supplementary Material online). Supplementary table S1, Supplementary Material online, lists all gene–drug interactions. (*b*) Changes in gene expression can confer drug resistance through a wide variety of mechanisms. Some of the identified genes have functional annotations that clearly suggest a mechanism of resistance (supplementary table S3, Supplementary Material online), while most currently lack functional annotation (25% of genes) or do not have a functional annotation related to antibiotic action (38% of genes).

For the majority of antibiotics tested, the screens revealed many pathways to resistance through gene expression changes. Sequencing approximately 2,400 drug resistant colonies identified over 200 gene–drug interactions where changing a gene’s expression level was repeatedly observed to increase resistance to an antibiotic (∼1,900 colonies with repeatedly observed interactions, 500 colonies with single-instance interactions; see supplementary table S1 and supplementary note, Supplementary Material online, for antibiotic-specific numbers. Statistical resampling indicated that for many antibiotics the number of gene–drug interactions identified had saturated at this level of sampling, but under some antibiotic treatments the number of nonoptimally expressed genes is likely to be greater still; supplementary fig. S3, Supplementary Material online). These changes in gene expression consisted of both overexpression and deletion (59% and 41% of genes, respectively; fig. 2*a*), and most conferred resistance to a single class of drug (93%). The majority of the genes identified have not been previously related to drug resistance to the best of our knowledge (83%, including 34 unannotated genes and 52 annotated with unrelated functions). When these genes were compared with screens for resistance to a variety of nonclinical toxins, we observed a statistically significant enrichment for the recall of genes previously associated with multistress resistance or large increases in MIC (supplementary fig. S4, Supplementary Material online) (Hirakawa et al. 2003; Soo et al. 2011). We also compared these results with previously reported transcriptional changes in *E. coli* strains that have evolved resistance in the laboratory (Suzuki et al. 2014), which revealed that our overexpression hits were significantly more likely to be upregulated than other genes in evolved drug resistant *E. coli* (*P* < 10^−6^), and deletion hits were significantly more likely to be downregulated in drug resistant *E. coli* (*P* < 0.03) (supplementary fig. S4, Supplementary Material online).

We detected multiple genes involved in the metabolism and transport of lipopolysaccharide, enterobactin, polyamines, and ubiquinone, suggesting that changes in the regulation of these molecules can contribute to resistance to specific antibiotics. Genes with annotation that suggested a relation to drug action encoded diverse functions, including modification of the cellular process affected by a drug (such as *sbmC* which inhibits DNA Gyrase and resists DNA-damaging drugs [Baquero et al. 1995; Nakanishi et al. 1998; Oh et al. 2001; Chatterji and Nagaraja 2002]), modification of cell permeability (such as *ompF* which facilitates entry of beta-lactam antibiotics into the cell [Nikaido 1989]), chemical modification of drugs (such as *ampC* which degrades beta-lactam antibiotics [Bergstrom and Normark 1979]), the activation of efflux and acid resistance systems (such as *marA* and *soxS* which confer multidrug resistance by transcriptional activation of drug efflux systems [Alekshun and Levy 1999; Martin and Rosner 2002]), and increased flux through a drug-inhibited pathway (such as *nudB*, which catalyzes the first committed step in folate biosynthesis [Suzuki and Brown 1974], resisting folate synthesis inhibitors) (fig. 2*b*; supplementary table S3, Supplementary Material online).

In every class of antibiotic, except cell-membrane-targeting drugs, many of these different modes of resistance were observed (fig. 2*b*). Even within general modes of resistance a remarkable diversity of mechanisms was observed. For example, four different strategies could increase pathway flux to resist folate synthesis inhibition: 1) Overproduction of a drug’s target enzyme (*folA*), 2) overproduction of a drug-insensitive replacement enzyme (*folM*), 3) overproduction of an upstream enzyme that controls total incoming flux (*nudB*), and 4) loss of enzymes that convert pathway metabolites to alternate products (*ΔfolX*, *ΔfolM*; note that *folM* can be overexpressed to resist trimethoprim or deleted to resist sulfonamides; supplementary tables S1 and S2, Supplementary Material online).

Nine genes were identified whose overexpression increased resistance to multiple antibiotics with diverse mechanisms. Together with the known multidrug resistance genes *marA* and *soxS* (Alekshun and Levy 1999; Martin and Rosner 2002), seven novel multidrug resistance genes of varied functions were identified. These include *gadW*, a transcriptional regulator of acid resistance; *bssR*, a regulator of biofilm; and *ddpF*, a putative component of an ABC transporter. The functional annotations of the remaining four multidrug resistance genes (*hemD*, *yhbT*, *gmr*, and *rbsR*; see supplementary table S1, Supplementary Material online) do not immediately suggest mechanisms of resistance.

Overexpression of antibiotic targets was found to confer resistance only in specific antibiotic classes. Genes encoding specific targets of drugs are of particular interest because if an antibiotic acts by inhibiting target activity, target upregulation or amplification may confer drug resistance; additionally, evolution may then act on a larger number of gene templates to drive the evolution of greater resistance (Andersson and Hughes 2009). However, in only 4 of 31 antibiotics was drug target overexpression observed to confer resistance. Most of these common Gram-negative antibiotics do not act on a single target gene, instead inhibiting a multiprotein complex, a family of related enzymes, or acting on a nonprotein target such as the cell membrane. Yet, target overexpression does not always confer resistance even for drugs with a single protein target, a result that is explained by recent research showing that target overexpression fails to confer resistance in two cases: First, for drugs that induce harmful target-catalyzed reactions, such as sulfonamides and quinolones, and second, for genes where overexpression alone incurs severe fitness costs, as is often observed for the targets of penicillins and cephalosporins (Meisel et al. 2003; Legaree et al. 2007; Palmer and Kishony 2014) (supplementary table S4, Supplementary Material online). Because drug–target interactions are often absent, and because the abundance of nonoptimal gene expression creates many expression-resistance interactions that do not represent drug targets, only a very small fraction of all expression-mediated paths to resistance represent target–drug interactions. For this reason, methods to identify a novel drug’s molecular target by screening for overexpression-mediated resistance, and possibly also knockdown-mediated sensitivity, may often be confounded by nonoptimal gene expression. This is supported by the frequent observation that resistance can result from expression changes in many nontarget enzymes in the target pathway or closely related pathways, and is best exemplified by sulfonamides, where resistance can develop from changes in the expression of enzymes both upstream and downstream of the target, but not the target itself.

The absence of a few known resistance mechanisms suggests limitations of these strain libraries. No components of the acrAB-tolC multidrug efflux pump were identified, likely because overexpression of a single component is alone unable to increase resistance. Loss-of-function mutations in *marR*, a repressor of *marA*, were also not observed here though they are known to confer multidrug resistance (Ariza et al. 1994; Toprak et al. 2012). This is likely due to a polar effect arising from the position of *marR* upstream of *marA* in a polycistronic operon, such that replacement of *marR* by a Kanamycin resistance cassette in the deletion library obstructs *marA* transcription. The mechanisms behind these known absences may be present more widely, suggesting that the present data set underestimates the number of genes that are nonoptimally expressed under antibiotic treatment.

### Antibiotics Perturb Fitness Functions according to a Log-Sensitivity Relation

The optimal expression levels of genes can dramatically shift under antibiotic treatment. The fitness effects of gene expression are composed of fitness benefits and costs of expression, which combine to generate a fitness versus expression function that is typically concave (because with increasing expression, benefits saturate and costs intensify) (Dekel and Alon 2005). The observation that changing the expression level of a gene can increase growth rate in the presence of antibiotics suggests that this fitness–expression function is maximized at different expression levels in drug-free compared with drug-treated environments. To directly observe how these fitness–expression functions are altered by antibiotics, we constructed experimentally controlled expression strains in which the gene of interest is deleted from the chromosome and resupplied on a plasmid under IPTG regulation (an empty plasmid was used for zero expression). The relationship between IPTG and transcription of a plasmid-borne gene was quantified using a *lacZ* reporter gene (note that the host strain is Δ*lacZYA*), revealing a monotonic relationship over IPTG doses up to 200 µM (supplementary fig. S5, Supplementary Material online), and furthermore this expression system was previously determined to behave robustly under partially inhibitory doses of mechanistically diverse antibiotics (Palmer and Kishony 2014).

**Fig. 3.**
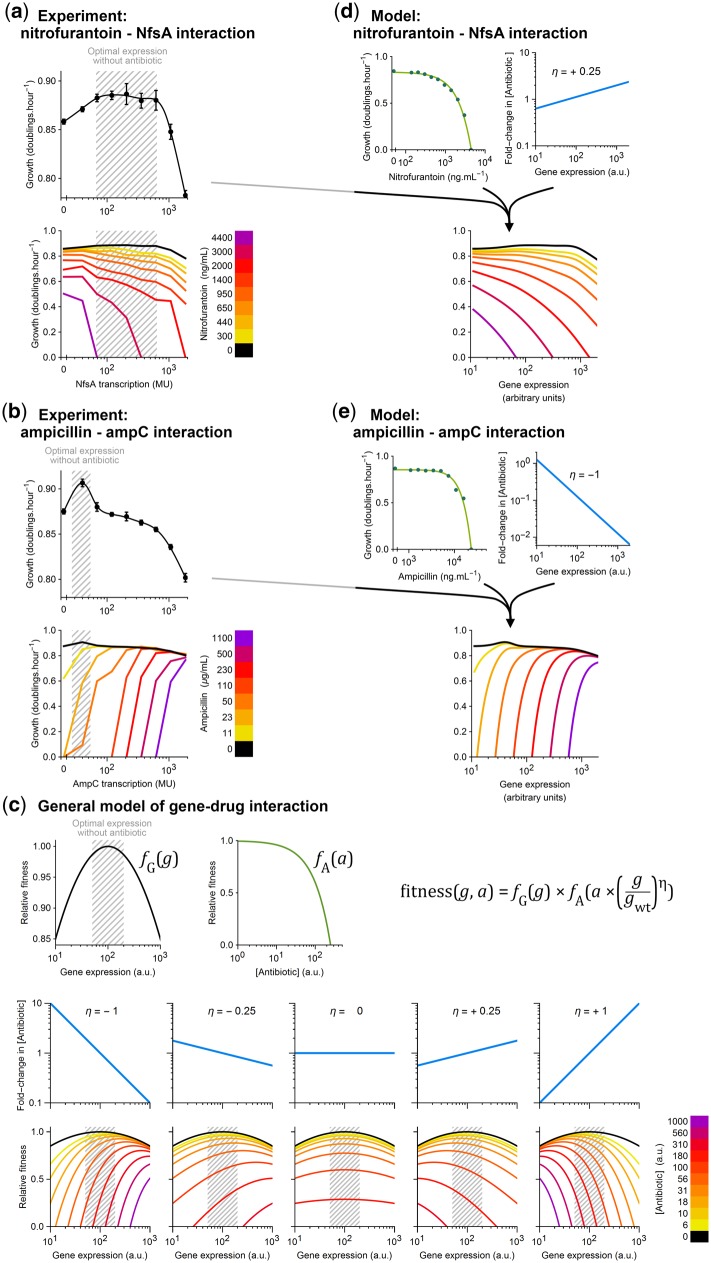
Antibiotic treatment alters optimal levels of gene expression. (*a, b*) *Escherichia coli* strains were constructed with experimentally controlled expression of *nfsA* or *ampC*, by deleting the respective gene from the chromosome and resupplying by plasmid an IPTG-inducible copy; zero expression was achieved with an empty plasmid (MU = Miller Units, quantified by LacZ assay; supplementary fig. S5, Supplementary Material online). Growth rates were measured as a function of gene expression using a sensitive bioluminescence assay (error bars are 95% confidence limits; *n* = 5 for *nfsA*, *n* = 10 for *ampC*). For each gene, growth is optimized in a particular expression range (gray shading). Fitness–expression functions were measured again at different doses of antibiotics that interact with these genes (nitrofurantoin–*nfsA*; ampicillin–*ampC*), revealing that antibiotic treatment shifts these functions so dramatically that expression levels which are optimal in the absence of antibiotic become lethally nonoptimal at high antibiotic concentrations. (*c*) A general model of how gene–drug interactions affect fitness is built from three parts: 1) *f*_G_ is the fitness effect of gene expression level *g*, 2) *f*_A_ is the fitness effect of antibiotic concentration *a*, and 3) gene–drug interaction is described by log-sensitivity η, where each 1% increase in gene expression affects a η% change in apparent antibiotic potency. Fitness as a function of gene expression and antibiotic dose is *f*_G_*× f*_A_. In general, *f*_G_ and *f*_A_ can be empirically determined; here for illustration, we use functions that resemble experimental observations: *f*_G_ is quadratic, *f*_A_ = 1 – *a*/*K* (*K* is a constant). For any log-sensitivity that substantially differs from zero (η ≤ –0.25 or η ≥ +0.25), a positive or negative change in gene expression can confer growth under antibiotic doses that are fully inhibitory when expression is at the antibiotic-free optimum (gray-shaded region). a.u., arbitrary units. (*d, e*) The observed antibiotic-induced changes in optimum gene expression levels for *nfsA* and *ampC* are quantitatively described by this model when using the measured relationships for *f*_G_ and *f*_A_, with η = +0.25 for *nfsA*–nitrofurantoin, and η = –1 for *ampC*–ampicillin.

We examined the *ampC*–ampicillin and *nfsA*–nitrofurantoin interactions as examples of overexpression- and deletion-mediated drug resistance. At fixed drug concentrations, bacterial growth rates were measured across IPTG gradients using a sensitive bioluminescence assay (Kishony and Leibler 2003). In the absence of drug, the costs and benefits of expressing these genes affect growth by only a few percent (fig. 3*a*). Indeed most genes in *E. coli*, including the majority of those identified here as nonoptimally expressed under drug treatment, have only modest effects on fitness when either deleted or strongly overexpressed (supplementary fig. S6, Supplementary Material online). However, as drug toxicity approaches lethal levels, expression levels that were optimal in the absence of drug become lethally nonoptimal in its presence, and the fitness–expression function is dominated by the effect of gene expression on drug susceptibility (fig. 3*b*).

These fitness landscapes are quantitatively explained by a simple model with a single parameter that relates gene expression to antibiotic potency. Bacterial fitness is modeled as the product of two functions: *f*_G_ is the effect of gene expression level *g* (considered in the absence of antibiotics), and *f*_A_ is the dose–response to antibiotic concentration *a* (considered at wild-type gene expression *g*_wt_), thus Fitness(*g*, *a*) = *f*_G_(*g*).*f*_A_(*a*). The gene–antibiotic interaction is introduced by a “log-sensitivity” η (also known as elasticity) whereby an *x*-fold change in gene expression causes an *x*^η^-fold change in antibiotic potency, so *a* becomes *a*.(*g*/*g*_wt_)^η^, and thus Fitness(*g*, *a*) = *f*_G_(*g*).*f*_A_(*a*.(*g*/*g*_wt_)^η^). This model defines family of fitness landscapes depending on *f*_G_ and *f*_A_, which can be experimentally measured, and a single free parameter η that quantifies interaction strength and sign (fig. 3*c*). Log-sensitivity η is modeled as being constant in the neighborhood of wild-type expression (fig. 3*c*, blue lines are straight), though sensitivity is likely to saturate (η→0) at very small and very large expression levels (far left and far right in fig. 3*c*). How far this relationship holds constant may depend on the nature of interaction: Enzymes that physically interact with a drug (e.g., trimethoprim and its target *folA*) can have constant sensitivity over a 100-fold range (Palmer and Kishony 2014), whereas an indirect regulatory effect, such as from a transcription factor, may saturate as soon as regulation of target genes saturates. Supplementary code, Supplementary Material online, is provided to implement this model. This simple model makes two predictions: First, for any gene whose expression level modifies the effective concentration of a toxin (i.e., whenever η ≠ 0), exposure to that toxin will change the optimal expression level of that gene; second, the expression level of such genes can have life-or-death consequences in the presence of the toxin, even if their expression level has only minor effects on fitness in the absence of stress. Together, these predictions explain why far from optimum gene expression is common under antibiotic treatment. Applying this model to experimentally measured fitness functions *f*_G_ and dose–responses *f*_A_ produces remarkably accurate matches to the observed interaction landscapes of *ampC*–ampicillin (at η = –1) and *nfsA*–nitrofurantoin (at η = +0.25) (fig. 3*d* and *e*). This model will not be precise in situations where η is not constant or where fitness is not as simple as the product of *f*_G_ and *f*_A_, but the general predictions are robust to quantitative deviations from these assumptions. Yet, an antibiotic-induced change in the optimum gene expression level does not guarantee nonoptimality, because natural regulatory systems may adaptively adjust expression under antibiotic treatment. We next investigated how well native regulation uses the potential for resistance in several examples of potent drug resistance genes.

### Drug Resistance Systems Are Often Poorly Utilized

**Fig. 4.**
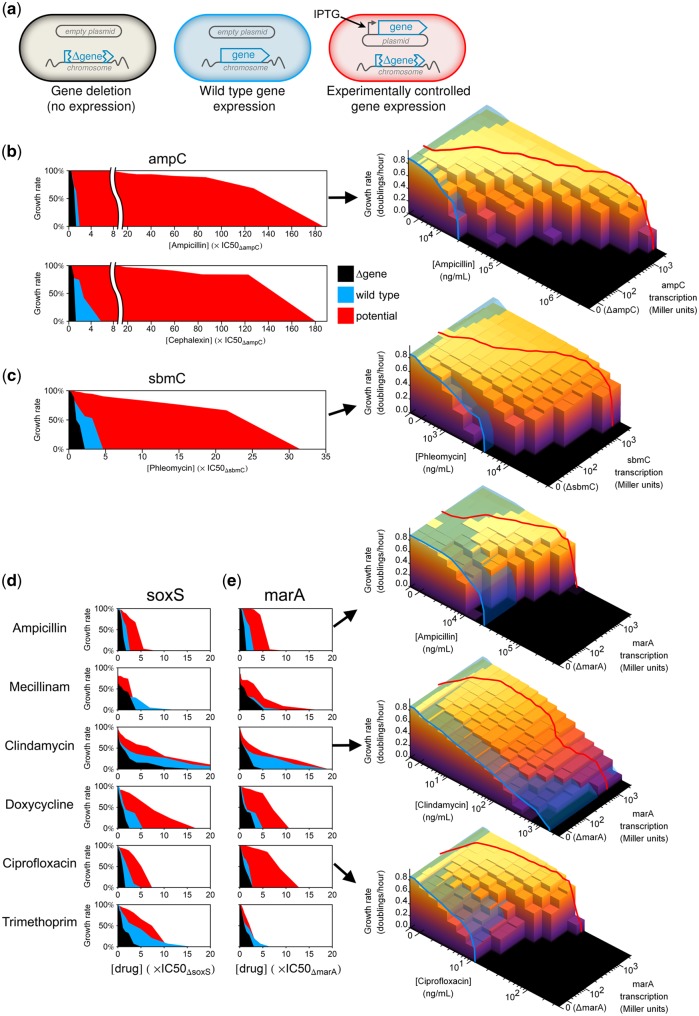
Intrinsic antibiotic resistance systems are often poorly utilized and hold unrealized potential for stronger resistance. (*a*) The optimality of a gene’s response to antibiotic treatment was investigated by comparing the drug susceptibility of three *Escherichia coli* strains: One lacking the gene (black), one with wild-type gene regulation (blue), and one where susceptibility can be measured over a range of experimentally controlled gene expression levels (red). (*b*) *ampC* encodes a potent beta-lactamase: Overexpression can confer a 100-fold increase in resistance to penicillins or cephalosporins. However, with wild-type regulation of *ampC* (blue) almost none of this potential for resistance (red) is used. Right: An empirical fitness landscape shows the growth inhibitory effect of an antibiotic at different levels of gene expression. Inhibition of the wild-type strain is illustrated by a transparent blue surface across the landscape; regions where the landscape is above the blue surface are levels of gene expression conferring antibiotic tolerance that is superior to wild type. A red line traces the gene expression response that maximizes growth at each drug dose. (*c*) *sbmC* encodes a DNA gyrase inhibitor whose overexpression confers resistance to the DNA-damaging drug phleomycin. However, a wild-type strain (blue) treated with phleomycin uses very little of the potential resistance offered by *sbmC* (red). (*d, e*) The use of *soxS* and *marA* was examined under treatment by nine different antibiotics (cefsulodin, nitrofurantoin, and phleomycin are not shown because marA and soxS had no effect on susceptibility; supplementary fig. S7, Supplementary Material online). For those drugs where experimental control of gene expression (red) increased resistance relative to gene deletion (black), the wild-type strain (blue) most often used only a fraction of the potential for drug resistance. Right: Selected examples of fitness landscapes of drug resistance versus *marA* expression. *marA* use is suboptimal in ampicillin and ciprofloxacin, and in clindamycin *marA* is only used effectively when growth is inhibited by more than 50%.

When a change in gene expression has the potential to confer antibiotic resistance, wild-type regulation might be using all, a fraction of, or none of this potential for resistance. To explore this, we focused on two strong drug-specific resistance genes, *ampC* and *sbmC*, and the two most broadly protective multidrug resistance genes, *marA* and *soxS*. To understand how well these drug resistance genes are natively utilized, we quantified to what extent wild-type resistance exceeds the resistance without the gene and how closely it approaches the maximum resistance at optimal gene expression. The maximal resistance was determined by constructing experimentally controlled expression strains and measuring their growth on 2D antibiotic-IPTG gradients in microtiter plates (as above, an empty plasmid was used for zero expression; fig. 4).

Wild-type regulation of the genes we tested was occasionally adequate, but in most cases achieved only a small fraction of the potential for resistance. Under treatment by either a penicillin or a cephalosporin, we observe that wild-type *ampC* expression levels provide negligible resistance relative to a *ΔampC* strain, despite the potential to confer 100-fold resistance when overexpressed (fig. 4*b*, supplementary fig. S7*b*, Supplementary Material online). Similarly, wild-type regulation of *sbmC* realizes only a small fraction of the potential for phleomycin resistance (fig. 4*c*, supplementary fig. S7*b*, Supplementary Material online). Expression of *marA* and *soxS* can confer resistance to several different antibiotics (fig. 4*d* and *e*). This potential for resistance is reasonably well used by the wild-type strain in the case of clindamycin (*marA* and *soxS*) and trimethoprim (*soxS* only) (unexpectedly, both synthetic antibiotics) but is poorly utilized in all other cases (fig. 4*d* and *e*;supplementary fig. S7*a*, Supplementary Material online). Past observations that activating mutations in the *mar* and *sox* operons confer multidrug resistance in clinical isolates is evidence that these systems are also poorly utilized during clinical treatment of *E. coli* infections, and thereby that the nonoptimality observed here is not an artifact of laboratory growth (Maneewannakul and Levy 1996; Koutsolioutsou et al. 2005). The observation here that some antibiotics trigger innate defenses and some do not has two implications. First, it affects the interpretation of genetic studies of drug resistance: If a mutation that changes a gene’s expression level confers resistance to drug A but not drug B, this does not necessarily mean the gene is only involved in drug A resistance, for it may simply have close to optimal regulation under drug B so that further mutational change is unhelpful. Second, it suggests that future study of the mechanistic or chemical determinants of this variation may be useful in designing antibiotics which preserve their efficacy by not inducing innate resistance mechanisms.

In summary, some but not all antibiotics could potentially be resisted by intrinsic stress response systems, and therefore these antibiotics are effective—at least before regulatory mutations arise—because native regulation does not fully employ those defenses.

### The “Intrinsic” Resistome Is Different from the “Latent” Resistome

**Fig. 5.**
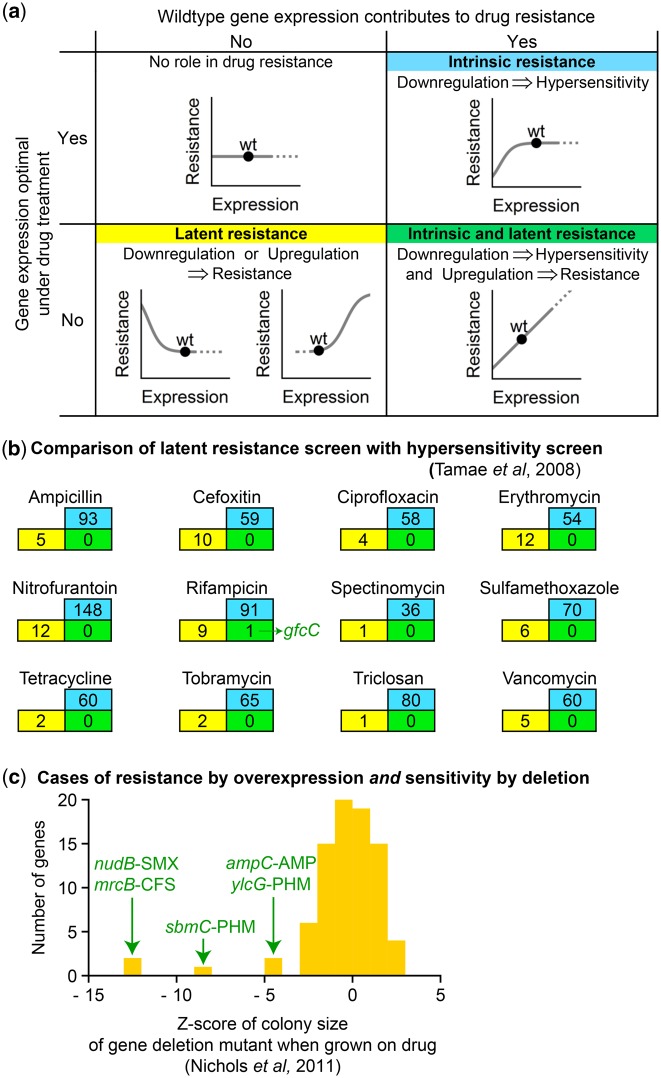
Drug resistance genes can be latent and/or intrinsic, depending on the optimality of wild-type gene expression under drug treatment. (*a*) The relationship between drug resistance and gene expression can take several different shapes, which illustrates a distinction between genes that contribute to resistance at wild-type expression—the intrinsic resistome—and genes with the potential to contribute to resistance if their expression is changed from wild type—the latent resistome. (*b*) The current screen for latent resistance genes (number of genes per antibiotic in yellow) has little overlap (number in green) with a published screen of “intrinsic resistance genes” (number in blue) (Tamae et al. 2008). (*c*) For 86 gene–drug interactions where overexpression conferred antibiotic resistance, a deletion mutant of the same gene has previously been grown in a subinhibitory dose of the same antibiotic and its colony size measured as a *Z*-score relative to the set of all viable gene deletion mutants in *Escherichia coli* (Nichols et al. 2011). In this comparison, five genes were observed to confer both resistance when overexpressed and strong hypersensitivity when deleted (green gene names). *marA* and *soxS* are not among these five, but show modest sensitivity to several antibiotics when deleted (*Z*-scores ≈ −1 to −2).

Our results suggest that resistance determinants can be classified into two categories (fig. 5*a*). The first category is the intrinsic resistome, which is revealed by drug hypersensitivity of gene knockout mutants (Tamae et al. 2008; Nichols et al. 2011). Here intrinsic means not only that the genes are present in the genome, but are functional: These genes make a measurable contribution to drug resistance at their native expression levels, whether or not it is optimal. The second category of resistance determinants contains genes where a change from native expression increases drug resistance. These genes must have been nonoptimally expressed under drug treatment, and can be considered the latent resistome. This group exists not only because resistance genes that directly protect against drug stress are often nonoptimally expressed, but also because any gene with nonoptimal expression under drug stress could manifest as a resistance gene. This occurs simply because optimizing gene expression under drug stress can improve survival; direct interaction with a drug or drug-inhibited pathway is unnecessary. In principle genes could belong to both the intrinsic and latent resistome, if native expression confers some resistance that is less than the maximum. However, comparing “latent resistance” genes with previous screens for the intrinsic resistome suggests that these categories have little overlap. Tamae et al. (2008) identified antibiotic-hypersensitive gene deletion mutants in *E. coli*; comparing their findings in the case of antibiotics also studied here reveals, as expected, that mutations are more likely to be harmful than beneficial (the intrinsic resistome is more populous than the latent resistome), and that it is rare for a gene to belong to both groups (fig. 5*b*). Nichols et al. (2011) measured the colony size of each *E. coli* gene deletion mutant when grown in subinhibitory antibiotic doses. Of the genes here identified to confer resistance to some antibiotic when overexpressed, only in five cases does deletion of that gene incur major growth defects when exposed to the same antibiotic (fig. 5*c*). Similarly, comparison with a screen for the intrinsic resistome by Girgis et al. (2009) identified only *ampC* as a gene exhibiting both intrinsic and latent resistance. These results emphasize the importance of the distinction between intrinsic and latent drug resistance genes, which are identified by different experimental designs and have different biological meaning.

## Discussion

An organism’s gene expression state, and gene regulatory machinery, evolves to adapt to the environmental conditions and perturbations that are regularly encountered in its ecological niche. However, when an organism encounters a novel or rare perturbation, the gene expression response may be far from perfect (Kalisky et al. 2007). Our results show that for nearly every antibiotic tested across diverse mechanisms, many microbial genes are have nonoptimal expression under antibiotic treatment with life-or-death consequences. A simple model was developed which allows the strength of a gene–antibiotic interaction to be evaluated by its log-sensitivity, which is independent of the size of a particular expression change. This model showed that under antibiotic treatment, even a weak linkage between gene expression and susceptibility will overwhelm the typically modest fitness effects of expression level (supplementary fig. S6, Supplementary Material online), such that changing expression away from the antibiotic-free optimum improves resistance (fig. 3). This effect is produced even by weak interactions, which might explain why gene expression changes in many aspects of bacterial physiology, most with no apparent connection to drug mechanism, can contribute to the evolution of antibiotic resistance (fig. 2*b*). We anticipate that potential clinical relevance will lie in the general phenomenon more so than in the specific mutations identified here, for two reasons. First, the majority of mutations have a magnitude of effect too small to individually confer clinically significant resistance (supplementary figs. S1 and S2, Supplementary Material online: most changes in zone of inhibition are <5 mm). Second, genes that indirectly influence drug susceptibility via an effect on cellular physiology may be environment dependent in their effect. The clinical relevance of numerous regulatory mutations contributing to drug resistance, perhaps each to a modest degree, is demonstrated by genome-sequencing of *Mycobacterium tuberculosis* isolates, which has identified dozens of mutations in intergenic regions and promoters that are each associated with antibiotic resistance (Sandgren et al. 2009; Farhat et al. 2013; Zhang et al. 2013).

The impact of nonoptimal gene expression on antibiotic susceptibility is variable between drugs. These differences can be visualized in terms of how far into a zone of inhibition drug-resistant colonies appear (fig. 1 and supplementary fig. S1, Supplementary Material online). Gene expression is not detectably nonoptimal under all clinically relevant antibiotics: For example, we identified no changes in gene expression that confer resistance to polymyxins. This does not imply that every gene’s expression is precisely tuned, because optimality merely means “as good as it gets,” and a functionally optimal state occurs when drug resistance is insensitive to gene expression or is only worsened by any large change (fig. 5*a*, top row). Some differences between drugs with similar mechanisms may be the consequence of an experimental design that emphasizes a low false discovery rate and therefore underestimates the number of true positives (supplementary fig. S3, Supplementary Material online). This is likely to cause an underestimation of the rate of cross-resistance conferred by individual mutations. Additionally, bacteria that evolve antibiotic resistance through the acquisition of multiple mutations are more likely to exhibit multidrug resistance. This is simply because with multiple mutations, there is a greater probability that any one mutation confers multidrug resistance, as has been observed in experimental directed evolution of antibiotic resistance (Toprak et al. 2012; Lázár et al. 2014).

Individual genes can show variable degrees of nonoptimality in different antibiotics. Multidrug resistance genes *marA* and *sox* have close to optimal induction in some drugs (e.g., Clindamycin) and far from optimal induction in others (e.g., Ciprofloxacin). Therefore the magnitude of resistance gained by *marA* overexpression is not only a property of how well the system protects against a given drug, but is strongly determined by the optimality or otherwise of wild-type regulation (fig. 4*b*). This case illustrates a general principle, whereby the magnitude of drug resistance conferred by a change in gene expression depends on two factors: First, the strength of the gene’s influence on drug potency; and second, the degree of nonoptimality of native expression under drug treatment. For example, the overexpression of a particular gene could confer a small increase in resistance when the gene is strongly protective but only slightly nonoptimal, or also because the gene is weakly protective but highly nonoptimal.

It is unclear what are the evolutionary, genetic, and biochemical reasons underlying these widespread shortcomings in responding to antibiotic stress, but they are likely to depend on the regulatory context and the nature of the stress. For example, in many bacterial species *ampC* is accompanied by a transcriptional activator, *ampR*, which senses the effects of beta-lactam treatment and induces *ampC*-mediated resistance (Lindquist et al. 1989; Dietz et al. 1997). However *E. coli* lacks *ampR*, and *ampC* expression instead serves to maintain normal cell morphology (Bishop and Weiner 1993; Henderson et al. 1997). Thus, whether a gene provides intrinsic or latent resistance can depend on the presence of regulators in its genomic context. Because antibiotic molecules, by their construction or natural selection, are unusually potent microbial toxins, we speculate that the *mar* and *sox* systems are poorly used under native regulation because such general-purpose xenobiotic defense systems may be insufficiently sensitive to respond to antibiotics at their generally low inhibitory concentrations. There are also examples of more elaborate reasons for nonoptimal gene expression: A drug or drug mixture may induce serious physiological imbalances between cellular components, outside the usual range of their regulation (Bollenbach et al. 2009) or may even induce a harmful regulatory response in drug resistance systems (Palmer et al. 2010). Most simply though, nonoptimal expression under antibiotic stress may result from a lack of evolutionary selection for adaptive responses to historically novel stresses. It would be interesting to see whether the extent of nonoptimal regulation varies across stresses and microbial species, and whether optimality is improved when a species responds to its natural stresses, particularly considering that many antibiotics are produced in natural microbial ecosystems.

The observation that specific resistance genes can be well used under some but not other antibiotics complicates the discovery of resistance genes by genetic screens: Overexpression may not increase resistance if the gene is already optimally induced by wild-type regulation (intrinsic resistance scenario in fig. 5*a*), and similarly, deletion of a resistance gene may not cause hypersensitivity if the gene is not expressed under treatment. Furthermore, for methods that seek to identify drug mechanism of action by identifying resistance-conferring mutations, or changes in drug susceptibility in the presence of engineered gene expression changes (Giaever et al. 1999), the present results indicate that such studies must be interpreted with caution for the possibly confounding effects of nonoptimal gene expression. Mutations that “optimize” capacity to survive antibiotic stress may involve breaking regulatory systems that are optimized for antibiotic-free growth, in order to massively overproduce resistance-conferring genes or entirely lose expression of susceptibility-conferring genes. As a consequence, the benefit of such mutations may rarely outlast the temporary antibiotic stress, leaving substantial fitness costs for growth in antibiotic-free environments.

Every imperfection in physiological adaptation provides an opportunity for evolutionary adaptation. Changes in gene expression constitute a large mutational target for the evolution of resistance to specific stresses because copy number alterations are abundant in bacterial populations (Andersson and Hughes 2009) and stresses themselves can induce mutations including copy number changes (Hastings et al. 2000; Bjedov et al. 2003; Galhardo et al. 2007), and—crucially—because gene expression is widely nonoptimal under stress, creating many opportunities for improvement. Thus, while bacteria can achieve antibiotic resistance by acquiring functional mutations and specific resistance mechanisms, we find that they can also draw on an unappreciatedly vast pool of mutations that contextually improve gene expression and activate latent defense mechanisms.

## Materials and Methods

### Strains and Media

All selection experiments and growth rate assays were performed in M63 minimal medium (2 g l^−1^ (NH_4_)_2_SO_4_, 13.6 g l^−1^ KH_2_PO_4_, 0.5 mg l^−1^ FeSO_4_•7H2O, adjusted to pH 7.0 with KOH) supplemented with 0.2% glucose, 0.1% casamino acids, 1 mM MgSO_4_, and 0.5 mg l^−1^ thiamine.

*Escherichia coli* strain BW25113 is the host for the KEIO gene deletion library (Datsenko and Wanner 2000; Baba et al. 2006). The strains of the KEIO gene deletion library (Baba et al. 2006) were grown individually in 384-well plates with 80 μl of Lysogeny broth per well, with incubation at 37 °C and shaking at 900 rpm for 24 h. All cultures were then collected in a single beaker and mixed, passed through 5-μm cellulose acetate filters to disrupt cell clumps, and frozen in aliquots at −80 °C in 10% glycerol. As the ASKA open reading frame (ORF) library of plasmids was supplied in the AG1 cloning strain (Kitagawa et al. 2005), the plasmids were purified and transformed in pools into the “wild-type” strain MG1655 *rph + * Δ*lacIZYA* (gift from K.E. Shearwin; constructed from BW30270 [CGSC7925] by precise deletion of *lacIZYA* [EcoCyc MG1655: 360527–366797] by recombineering) to minimize artifacts arising from the poor health of the cloning strain. The *lacIZYA* deletion engineered into this MG1655 strain allows the use of IPTG to exclusively induce plasmid-based expression without additional fitness effects due to induction of the *lac* operon. No such alterations were required for the deletion library as BW25113 has only minor perturbations relative to wild-type MG1655, and IPTG was not required to induce a change in gene expression. The AG1 strains of the ASKA library were grown by the same protocol as described for the KEIO library, and the chloramphenicol resistance and ORF-encoding pCA24N plasmids were isolated as a pooled mixture from each 384-well plate by a QIAGEN Spin Miniprep kit. Plasmid pools were transformed into MG1655 *rph*^+^ Δ*lacIZYA* by the one-step protocol of reference (Chung et al. 1989). To enrich for plasmid transformed cells, liquid cultures of transformed *E. coli* (∼10^9^ cfu) were inoculated into 10 ml of M63 minimal medium with 30 μg ml^−1^ chloramphenicol and were incubated overnight at 37 °C with shaking at 300 rpm. All transformant pools were added to a single flask, mixed, and frozen in aliquots at −80 °C in 10% glycerol. Transforming the complete overexpression library into a fresh background has the benefit that in the drug diffusion screen, each ORF-expression plasmid is represented in the zone of selection not by multiple descendants of a common transformation event, but by descendants of many transformation events (typically no more than one descendant of each transformation within a 3-mm zone of mutant selection), conferring confidence that resistance, if observed in multiple colonies with the same plasmid, is conferred by the plasmid rather than the background. A wild-type reference for the ASKA library was constructed by transforming MG1655 *rph*^+^ Δ*lacIZYA* with a pCA24N plasmid encoding yellow fluorescent protein (*yfp*), but with the promoter deleted (pCA24N-ΔpT5lac-*yfp*).

Growth rate assays in figures 3 and 4 were conducted in BW25113 background; in these figures, “wild type” refers to BW25113, “gene deletion” refers to the member of the KEIO deletion library lacking the gene of interest (BW25113 *gene*::Kan^R^), where the Kanamycin resistance cassette has been excised by FLP recombinase from a temperature-sensitive helper plasmid, yielding a strain BW25113 *gene*::FRT (Datsenko and Wanner 2000). Excision of kanamycin resistance and loss of the helper plasmid, by colony purification at 43 °C, were verified by testing for loss of antibiotic resistances. Controlled gene expression was produced by complementing a gene deletion strain with a pCA24N plasmid with IPTG-inducible expression of the deleted gene; these plasmids were obtained from the ASKA library and the ORFs were sequenced to confirm gene identity (Kitagawa et al. 2005). As *lacIZYA* is deleted in BW25113, IPTG does not incur fitness costs for *lac* operon production (Stoebel et al. 2008), and graded induction is possible without the LacY permease, that would otherwise cause all-or-none induction of LacI-regulated promoters (Novick and Weiner 1957; Choi et al. 2008). Regarding the drug diffusion assay (described below), this removal of “all-or-none” induction is necessary for different IPTG concentrations to induce different levels of gene expression, rather than inducing different fractions of the population to be “off” or “all on.” For consistency, both wild type and gene deletion strains were transformed with pCA24N-ΔpT5lac-*yfp*. All strains were then transformed with plasmid pCSλ which encodes a constitutively expressed bacterial bioluminescence operon (Kishony and Leibler 2003).

Drug solutions were made from powder stocks (from Sigma Aldrich unless specified otherwise: amikacin, A1774; ampicillin, A9518; azithromycin, Tocris 3771; bleomycin, Selleck S1214; carbenicillin, C1613; cephalexin, C4895; cefoxitin, C4786; cefsulodin, C8145; ciprofloxacin, 17850; chloramphenicol, C0378; clindamycin, Indofine C0117; colistin, C4461; doxycycline, D9891; erythromycin, Fluka 45673; isopropyl β-d-1-thiogalactopyranoside, Omega Bio-Tek AC121; kanamycin, K1876; lomefloxacin, L2906; mecillinam, 33447; nalidixic acid, N3143; nitrofurantoin, N7878; ortho-nitrophenyl-β-galactoside, N1127; penicillin G, Fluka 13750; phleomycin, P9564; polymyxin-B, P0972; rifamycin SV, Biochemika 83909; spectinomycin, S9007; spiramycin, S9132; streptomycin, S6501; sulfacetamide, S8627; sulfamethoxazole, S7507; tetracycline, 87128; tobramycin, T4014; triclosan, TCI America T1872; trimethoprim, T7883; vancomycin, V8138). Drug and IPTG gradients were made by serial dilution in M63 medium.

### Pooled-Library Drug Diffusion Assay

Frozen aliquots of deletion library host (BW25113), or pooled deletion library, or overexpression library host (MG1655 *rph*^+^ Δ*lacIZYA* pCA24N-ΔpT5lac-*yfp*), or pooled overexpression library, were thawed, and 10^7^ cells were spread by glass beads on wet 10-cm Petri dishes containing 25 ml of 1.5% agar M63 minimal media; with 15 or 150 μM IPTG only when plating the pooled overexpression library. Based on the typical sizes of zones of mutant selection (a 3-mm annulus at diameter of 30 mm ≈ 200-mm^2^ area), and the inoculum, we calculate that each gene expression mutant is typically represented by up to 100 colonies in the zone of mutant selection (depending on how far past the edge of the zone of inhibition they grow); therefore, this inoculum provides each expression mutant with a chance of identification if it confers drug resistance. Plates were briefly dried in a biosafety cabinet before an aliquot of antibiotic was pipetted in the center of the plate. Supplementary table S5, Supplementary Material online, lists the concentrations and volumes of each antibiotic solution used in this assay. Plates were incubated at 37 °C for 48 h before being photographed by a custom plate imager (Chait et al. 2010). Plates treated with sulfacetamide and sulfamethoxazole were instead incubated for 1 week due to the slow growth of drug-resistant colonies (these grow at usual speed when transferred onto drug-free agar); in all other drugs, resistant colonies either appeared within 48 h or were not apparent even after 1 week. Up to 48 drug-resistant colonies per plate (not including wild-type reference plates) were viewed in a Nikon SMZ-745T stereomicroscope, picked by a flame-sterilized 0.25-mm nichrome wire, and streaked on selective agar: 50 μg ml^−1^ of kanamycin for the gene deletion library and 30 μg ml^−1^ of chloramphenicol for the gene overexpression library. Streaked plates were incubated at room temperature for several days, a single colony from each plate was inoculated into a liquid culture of selective Lysogeny broth in a 96-well microtiter plate. Microtiter plates were incubated overnight at 37 °C with shaking at 900 rpm, and glycerol was added to each well to a final concentration of 15%. Microtiter plates were stored frozen at −80 °C. Genes in the overexpression library that confer drug resistance were identified by sending bacterial cultures to GENEWIZ to Sanger sequence the ORF of the pCA24N plasmid using the primer ASKAseqLF (CACCATCACCATCACCATACG). Gene deletions in the KEIO library that confer drug resistance were identified by Sanger sequencing the products of a 2-step hemi-nested polymerase chain reaction (PCR) that amplified a portion of chromosome adjacent to the Kanamycin resistance cassette that replaces each deleted gene. Both PCR steps used 20-μl reactions with 2 units of OneTaq DNA Polymerase (New England Biolabs M0480), 200 nM of each primer (Integrated DNA Technologies), and 200 μM of each dNTP (New England Biolabs N0447). The first PCR was inoculated with 1 μl of liquid bacterial culture, and used the three primers KEIOseq1 (TGAAGTTCCTATTCCGAAGTTCCTATTCTC), CEKG2C (GGCCACGCGTCGACTAGTACNNNNNNNNNNGATAT), and CEKG2D (GGCCACGCGTCGACTAGTACNNNNNNNNNNACGC) in the following reaction cycle: First 5′ at 95 °C; 6 cycles of 30″ at 95 °C, 30″ at 42 °C (lowering by 1 °C per cycle), 3′ at 68 °C; then 24 cycles of 30″ at 95 °C, 30″ at 45 °C, 3′ at 68 °C; and finally 5′ at 68 °C. The second PCR was inoculated with 0.5μl per well of the completed first PCR, and used the primers KEIOseq3 (TCGAAGCAGCTCCAGCCTAC) and CEKG4 (GGCCACGCGTCGACTAGTAC) in the following reaction cycle: 30 cycles of 30″ at 95 °C, 30″ at 56 °C, 3′ at 68 °C; and finally 5′ at 68 °C. Products of this final PCR were sent to GENEWIZ for sequencing by the KEIOseq3 primer. Sequences were aligned with BlastN to the *E. coli* MG1655 genome (NC_000913.2) to determine gene identity (Altschul et al. 1990; Blattner et al. 1997). Alignments that started more than 100 nucleotides from the expected start of alignment were discarded: Gene overexpression sequences should align shortly after the start codon of an ORF; gene deletion sequences should align shortly after the stop codon of an ORF.

### Drug Diffusion Assay Secondary Validation

Single mutant strains (gene deletion or gene overexpression) were inoculated at 1:500 frequency into the matching wild-type strain (for gene deletion, BW25113; for gene overexpression, MG1655 *rph*^+^ Δ*lacIZYA* pCA24N-ΔpT5lac-*yfp*). Each mixed culture of wild type and mutant was plated on a Petri dish and treated with a diffusing point source of antibiotic, precisely as performed in the primary screen. From each plate, four drug-resistant colonies growing inside the zone of inhibition were picked with a flame-sterilized 0.25-mm nichrome wire, and streaked onto selective agar. Gene deletion mutants were tested by streaking resistant colonies onto Kanamycin, on which only the gene deletion mutants are proficient for growth, because wild-type BW25113 is not Kanamycin resistant. Therefore, growth or no-growth on Kanamycin was sufficient to identify when selected colonies were the “spike-in” gene deletion mutant or the wild type. Gene overexpression mutants were streaked onto Chloramphenicol, on which both wild type and mutants are proficient for growth. Restreaked colonies were tested by colony PCR, inoculating a small quantity of colony into a reaction that amplifies the promoter present in the ORF-expressing plasmids, and not the control plasmid present in wild-type reference wherein the promoter had been deleted. Primers were ATTTGCTTTGTGAGCGGATAAC and ATCAACAGGAGTCCAAGCTCAG, used with the following reaction: 30 cycles of 30″ at 95 °C, 30″ at 51 °C, 60″ at 68 °C; and finally 5′ at 68 °C. Reaction products were visualized by agarose gel electrophoresis to distinguished wild-type colonies from ORF-expressing colonies.

### Growth Rate Assay

Bacterial growth rates were measured by bioluminescence precisely as described by Palmer and Kishony (2014). Notably, the pCSλ plasmid confers constitutive bioluminescence that allows cell densities in growing cultures to be precisely measured over many orders of magnitude by photon counting (Kishony and Leibler 2003). Growth rate is thus taken from the steepest slope of the logarithm of photon counts over a 6-h time span of constant growth, corresponding to five doublings in a healthy culture. The slope of the logarithm of photon counts is unaffected by changes in luminescence per cell, such as might result from antibiotic treatments or changes in gene expression (Kishony and Leibler 2003). Note that because the small inoculum causes growth to enter the measurable regime only after several hours of growth, and because the sensitivity of the method allows growth to be measured over many hours of exponential growth, it is improbable that transient kinetics of induction following first exposure to IPTG should influence steady-state growth rates. The IPTG-inducible gene expression system utilized in these experiments (figs. 3 and 4) has previously been characterized under partially inhibitory doses of several mechanistically distinct antibiotics, and it was found to be robust to antibiotic perturbation (Palmer and Kishony 2014). Specifically, the quantitative relationship between IPTG and the activity of a LacZ reporter gene was not substantially altered by antibiotic inhibition of growth to the range of 50–80% of uninhibited growth, with the exception of a cell wall antibiotic (mecillinam) under which IPTG appeared to enter the cell more readily. In figure 3*a* the growth trend line is a B-spline, and in figure 3*b* experimentally measured points are connected by straight lines.

### Beta-Galactosidase Assay

Kinetic beta-galactosidase (LacZ) assays were performed precisely as described by Palmer and Kishony (2014), taking 20 μl of culture for lysis and assay.

## Supplementary Material

Supplementary Data
