# Supplementary material for: Nonoptimal Gene Expression Creates Latent Potential for Antibiotic Resistance
Source: Mol Biol Evol. 2018 Aug 28;35(11):2669–84. doi: 10.1093/molbev/msy163 (PMC6231494; doi:10.1093/molbev/msy163)
Supplement: Supplementary Data [file msy163_supp.zip › msy163_Supp/Kishony_SI.pdf]

Supplementary Information for
Non-optimal gene expression creates latent potential for antibiotic resistance

Figure S1. Pooled diffusion-based selection for changes in gene expression that confer antibiotic resistance. 10^7 colony forming units of a clonal wildtype strain or a pooled library of gene deletion or gene over-expression mutants were plated on M63 glucose minimal media agar. An aliquot of antibiotic was added to the center and plates were incubated for 48 hours at 37°C before imaging. Images here show the presence of gene expression mutants with resistance to antibiotics that act upon: **a**, cell wall synthesis; **b**, the cell membrane; **c**, transcription; **d, e**, translation; **f**, DNA synthesis; **g**, free radical production. Plates treated with sulfacetamide and sulfamethoxazole were incubated for 1 week before imaging due to the slow growth of sulfonamide resistant colonies.

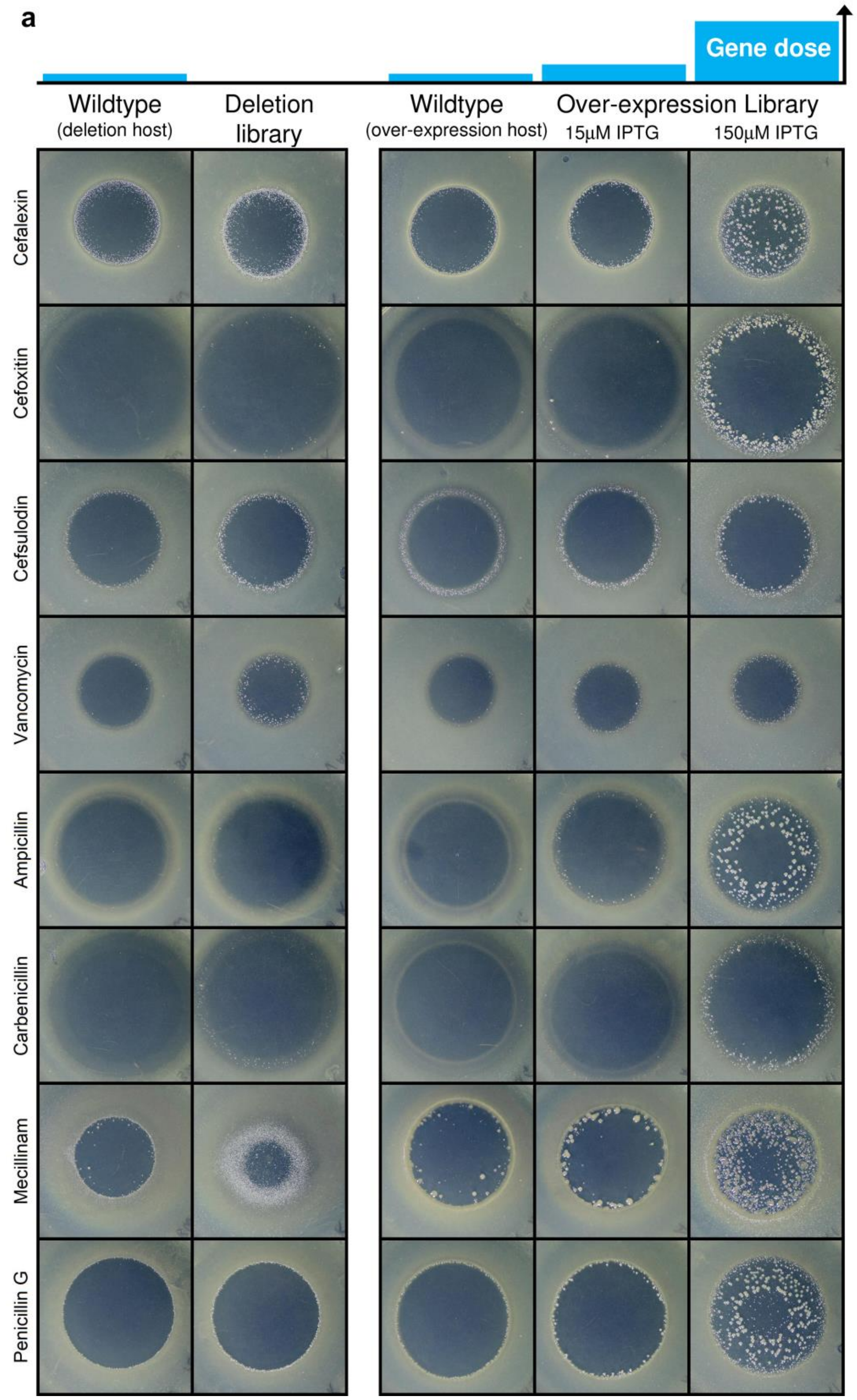

Figure S1 (continued).

b

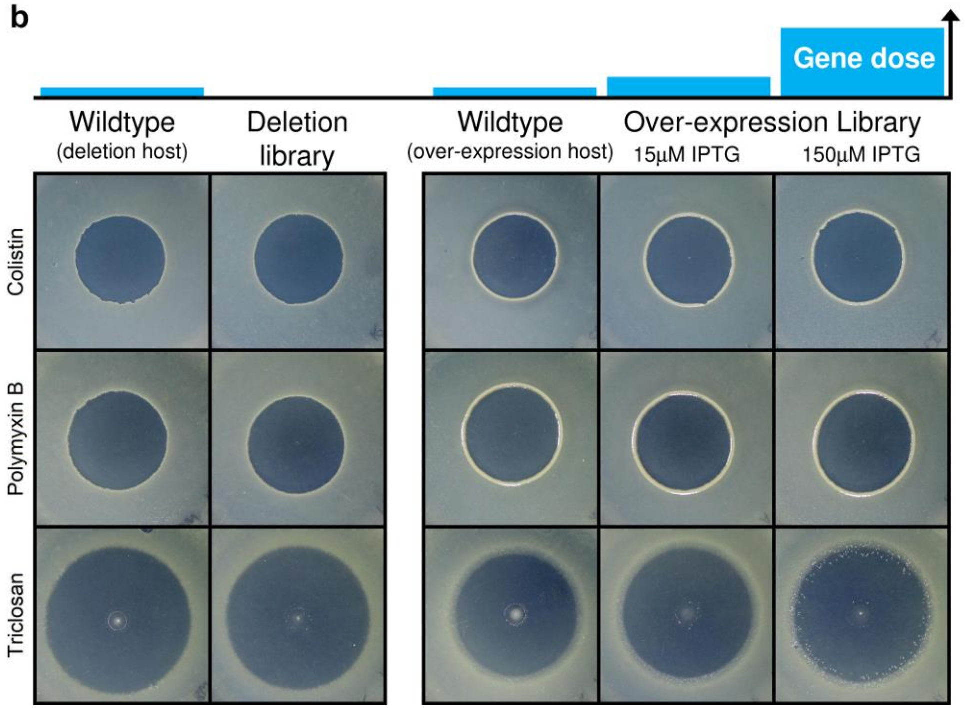

c

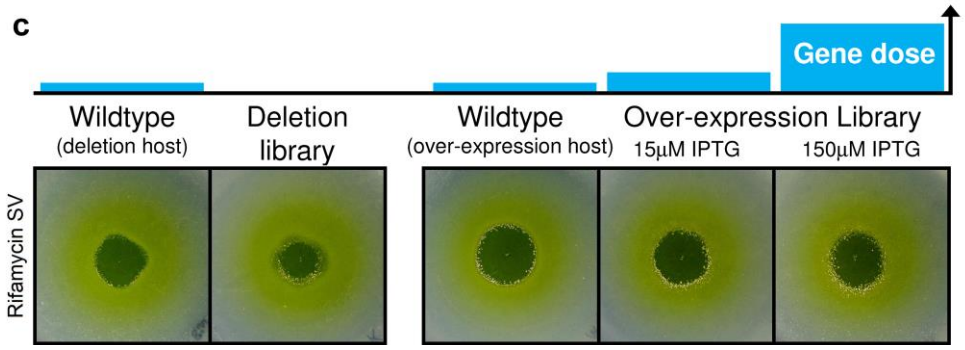

Figure S1 (continued).

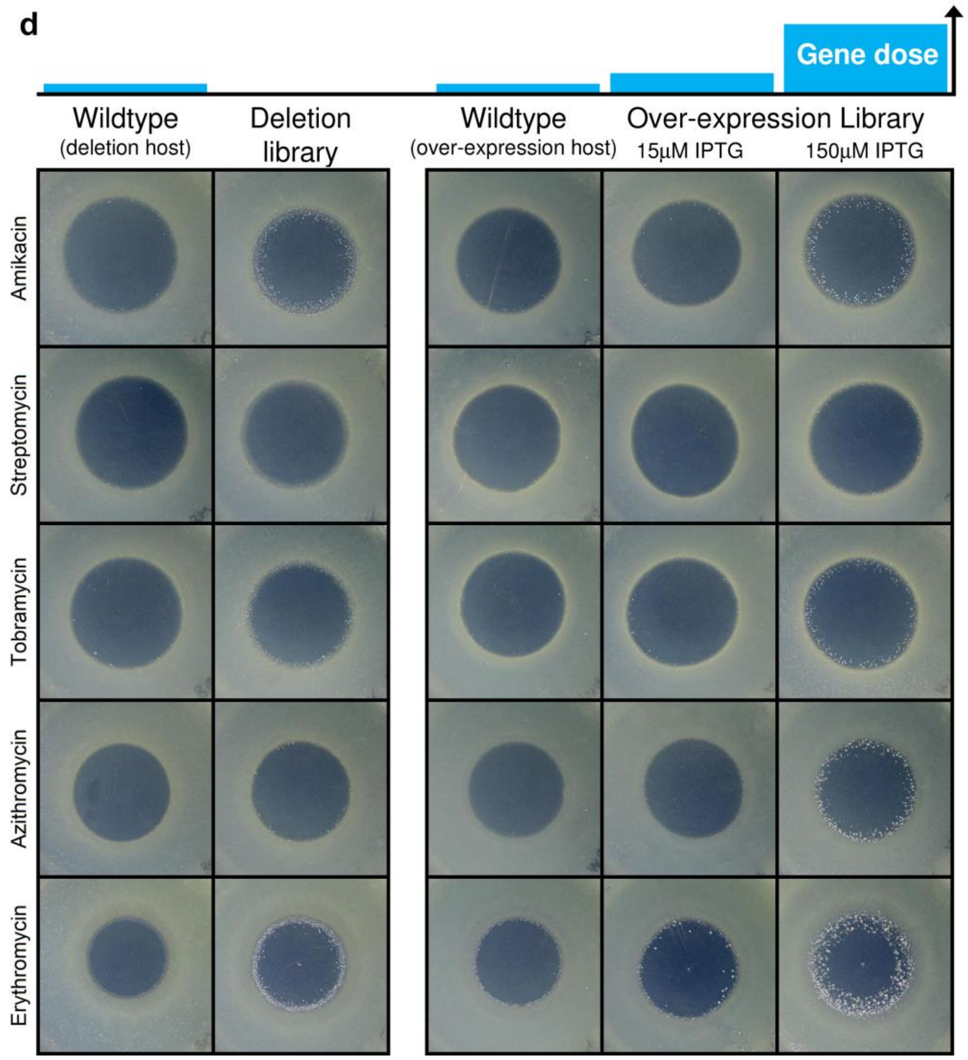

Figure S1 (continued).

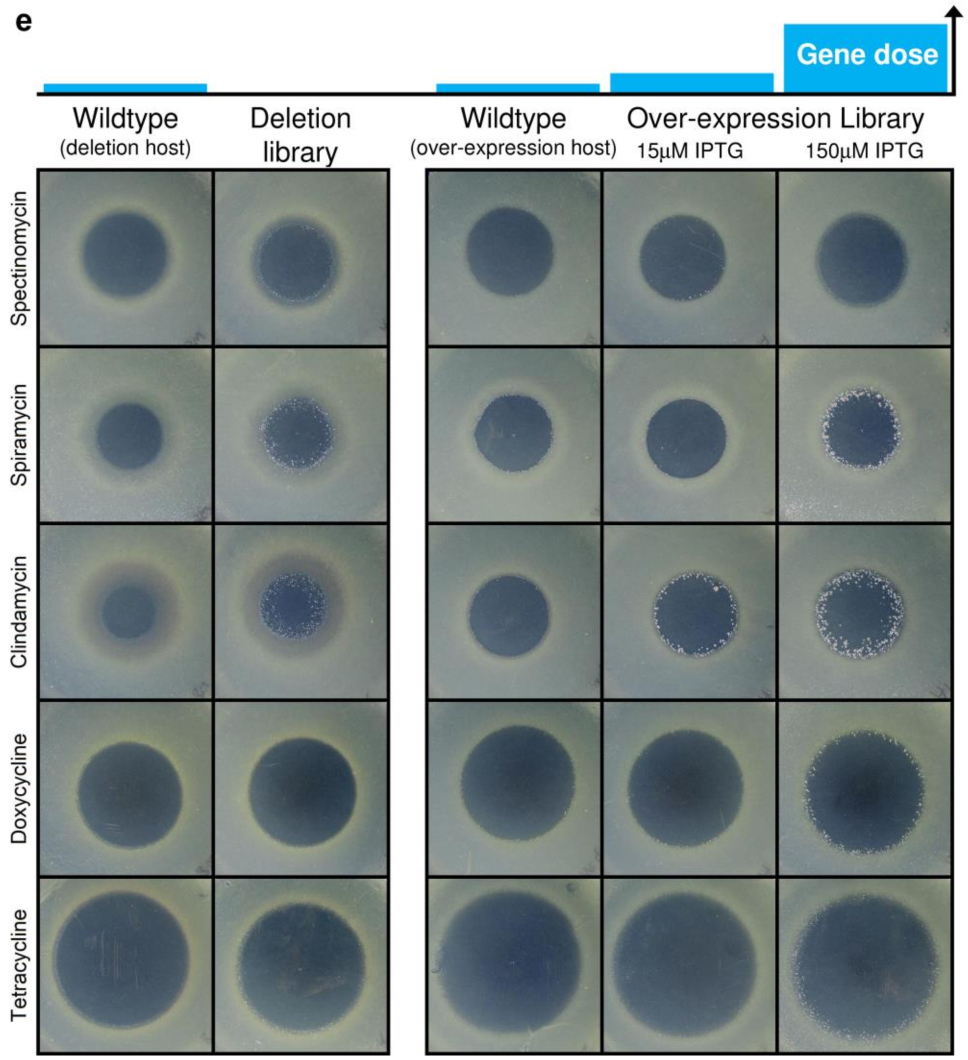

Figure S1 (continued).

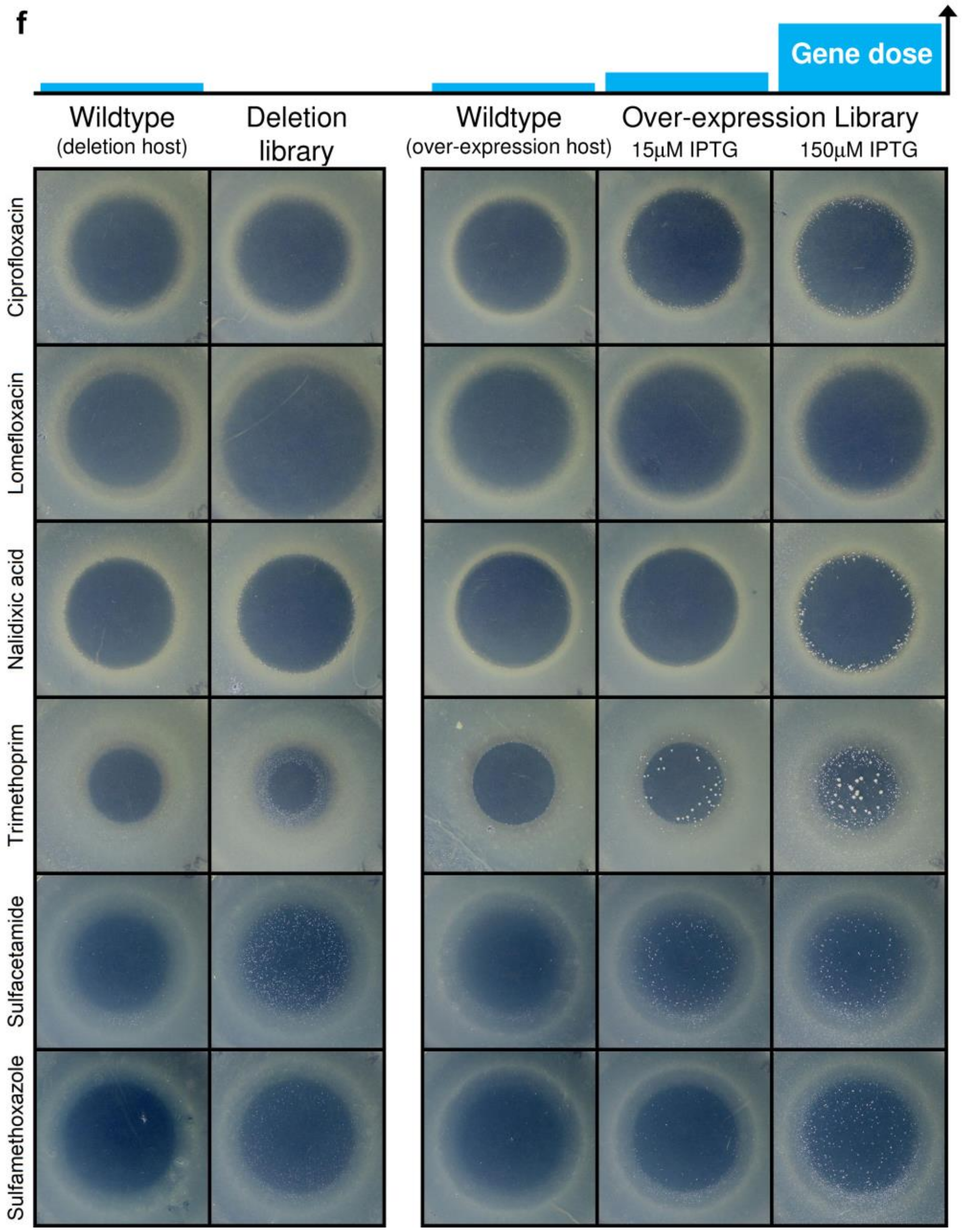

Figure S1 (continued).

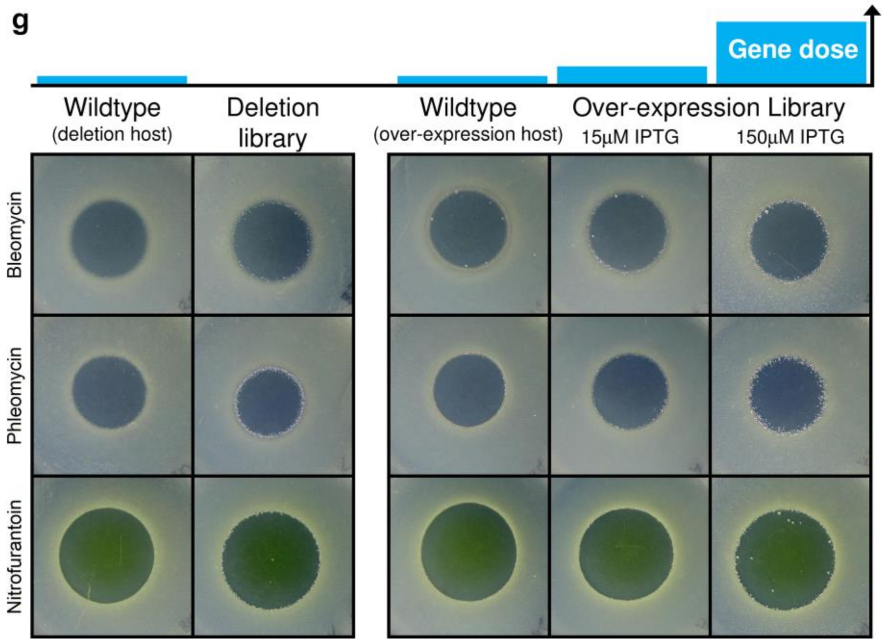

Figure S1 (continued).

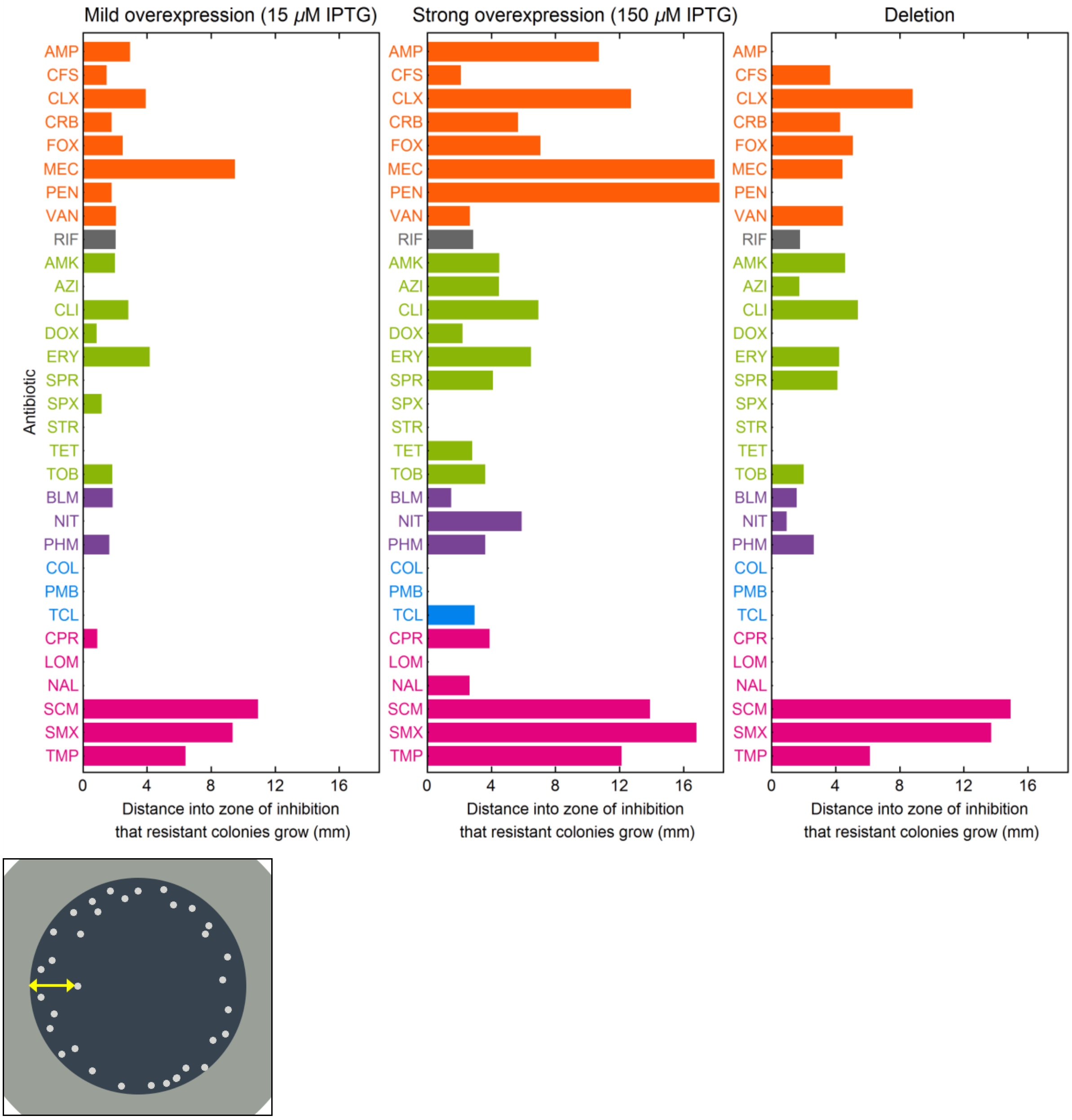

Figure S2. Non-optimal gene expression has varying quantitative effects on the level of antibiotic resistance. For each petri dish in the pooled-library drug diffusion assay (Fig. S1), the radial distance was digitally measured (Adobe Photoshop Ruler tool, calibrated against diameter of petri dish) from the most drug resistant colony, being that which extends the farthest into the center of the plate, to the nearest boundary of the zone of growth inhibition (yellow arrow in schematic). Antibiotics are grouped and colored by their target process (cell wall synthesis, orange; transcription, gray; translation, green; free radical production, purple; cell membrane, blue; DNA synthesis, magenta).

Table S1. Changes in gene expression that increase antibiotic resistance. Gene-drug interactions from Figure 2 are tabulated with gene functions curated from the Ecocyc database (Keseler et al, 2011)

Change in gene expression	Gene name	Drugs resisted	Gene function	Previously associated with drug resistance?	Notes
Overexpression	hemD	AMK, MEC, PHM, TOB	uroporphyrinogen III synthase	N	
Overexpression	yhbT	AMK, PHM, TOB	predicted lipid carrier protein	N	
Overexpression	ampC	AMP, CLX, CRB, FOX, PEN	β-lactamase	Y	(Linstrom et al, 1970)
Overexpression	marA	AMP, BLM, CLI, CLX, CPR, DOX, ERY, NAL, PEN, TET	MarA DNA-binding transcriptional dual regulator	Y	(Cohen et al, 1993)
Overexpression	nanA	AMP	N-acetylneuraminate lyase	N	nanA is the first enzyme in pathway for degradation of sialic acid (Vimr & Troy, 1985).
Overexpression	soxS	AMP, AZI, BLM, CLI, CPR, DOX, ERY, FOX, NAL, PEN, SPR, TET, TMP	SoxS DNA-binding transcriptional dual regulator	Y	(Amabile-Cuevas & Demple, 1991)
Overexpression	yidF	AMP, CLX, PEN	predicted DNA-binding transcriptional regulator	N	
Overexpression	gadW	AZI, BLM	GadW DNA-binding transcriptional dual regulator	N	gadW is a regulator of Glutamic acid decarboxylase (GAD) acid resistance system (Tucker et al, 2003).
Overexpression	rpmH	AZI, ERY, SPR	50S ribosomal subunit protein L34	N	Overexpression of rpmH decreases the production of polyamine (Panagiotidis et al, 1995).
Overexpression	ydeO	AZI	YdeO DNA-binding transcriptional dual regulator	N	ydeO activates transcription of acid resistance genes (Masuda & Church, 2003).
Overexpression	yeaH	AZI, ERY, SPR	conserved protein	N	
Overexpression	sbmC	BLM, PHM	DNA gyrase inhibitor	Y	Overexpression of sbmC confers resistance to mitomycin C (Wei et al, 2001) and microcin B17 (Baquero et al, 1995).
Overexpression	yjcH	BLM	conserved inner membrane protein	N	

Change in gene expression	Gene name	Drugs resisted	Gene function	Previously associated with drug resistance?	Notes
Overexpression	ddpF	CFS, CLX, MEC, PHM	putative ATP-binding component of an ABC transporter	N	While ddpF overexpression resists CFS, CLX, MEC, PHD, ddpF deletion resists AMK
Overexpression	degQ	CFS	serine endoprotease	N	
Overexpression	mrcB	CFS	Murein polymerase (PBP1b)	Y	mrcB and mrcA are the primary targets of Cefsulodin (Kong et al, 2010).
Overexpression	nlpE	CFS, CRB, PEN	outer membrane lipoprotein	N	
Overexpression	adk	CLI	adenylate kinase	N	
Overexpression	cadA	CLI	lysine decarboxylase 1	N	cadA is part of the lysine-dependent acid resistance system 4 (Takayama et al, 1994).
Overexpression	hflX	CLI, ERY	GTPase associated with the 50S subunit of the ribosome	N	
Overexpression	lepA	CLI	elongation factor 4	N	
Overexpression	proQ	CLI	RNA chaperone	N	
Overexpression	ybiT	CLI	putative ATP-binding component of an ABC transporter	N	
Overexpression	yheS	CLI	putative ATP-binding component of an ABC transporter	N	
Overexpression	aroB	CLX	3-dehydroquinate synthase	N	
Overexpression	bssR	CLX, CPR, FOX, NIT	regulator of biofilm formation	N	
Overexpression	gmr	CLX, FOX, NIT, PEN	modulator of RNase II stability	N	
Overexpression	gntT	CLX	gluconate transporter	N	
Overexpression	nanK	CLX, FOX	*N*-acetylmannosamine kinase	N	

Change in gene expression	Gene name	Drugs resisted	Gene function	Previously associated with drug resistance?	Notes
Overexpression	rcsD	CLX	Regulator of capsular polysaccharide synthesis	N	
Overexpression	rluA	CLX	23S rRNA and tRNA pseudouridine synthase	N	
Overexpression	yccT	CLX, PEN, VAN	conserved protein	N	
Overexpression	yjjQ	CLX, PEN	predicted DNA-binding transcriptional regulator	N	yjjQ is associated with methylglyoxal sensitivity (Kim et al, 2007).
Overexpression	rbsR	CPR, FOX, PEN	'Ribose Repressor' DNA-binding transcriptional repressor	N	
Overexpression	baeR	CRB	BaeR transcriptional regulator	Y	baeR activates the MdtABC drug efflux system (Nagakubo et al, 2002).
Overexpression	iap	CRB, PEN	alkaline phosphatase isozyme conversion protein	N	
Overexpression	amiA	ERY, SPR	N-acetylmuramoyl-L-alanine amidase 1	N	
Overexpression	appY	ERY	'acid phosphatase' DNA-binding transcriptional activator	N	
Overexpression	rluE	ERY, SPR	23S rRNA pseudouridine synthase	N	
Overexpression	rng	ERY	ribonuclease G	N	
Overexpression	btuE	FOX	thioredoxin/glutathione peroxidase	N	
Overexpression	ycgZ	FOX, PEN	predicted protein	N	ycgZ is a member of the cold shock stimulon (Polissi et al, 2003).
Overexpression	dxs	MEC	1-deoxyxylulose-5-phosphate synthase	N	Dxs is the first enzyme in the methylerythritol phosphate pathway of isoprenoid biosynthesis (Sprenger et al, 1997).
Overexpression	gcvA	MEC	'Glycine cleavage A' DNA-binding transcriptional dual regulator	N	
Overexpression	glnB	MEC	nitrogen regulatory protein P-II 1	N	

Change in gene expression	Gene name	Drugs resisted	Gene function	Previously associated with drug resistance?	Notes
Overexpression	glyQ	MEC	glycyl-tRNA synthetase, α subunit	N	
Overexpression	mrdA	MEC	peptidoglycan synthetase (PBP2)	Y	mrdA is the primary target of Mecillinam (Kong et al, 2010).
Overexpression	rplJ	MEC	50S ribosomal subunit protein L10	N	
Overexpression	rutR	MEC, PEN	'pyrimidine utilization, rut repressor' DNA-binding transcriptional dual regulator	N	
Overexpression	trpR	MEC	tryptophan transcriptional repressor	N	
Overexpression	yehK	MEC	predicted protein	N	
Overexpression	ygbE	MEC	conserved inner membrane protein	N	
Overexpression	ycjR	NIT	predicted component of the SdsRQP multidrug efflux pump	Y	(Dinh et al, 1994)
Overexpression	yibF	NIT	glutathione transferase-like protein	N	
Overexpression	eptB	PEN, VAN	phosphoethanolamine transferase	N	eptB modifies lipopolysaccharide (Reynolds et al, 2005).
Overexpression	nadK	PEN	NAD kinase	N	
Overexpression	cpdA	PHM	cAMP phosphodiesterase	N	Overproduction of cpdA confers acid resistance (Barth et al, 2009).
Overexpression	frsA	PHM	fermentation/respiration switch protein	N	
Overexpression	metB	PHM	O-succinylhomoserine lyase / O-succinylhomoserine(thiol)-lyase	N	
Overexpression	pdhR	PHM	pyruvate dehydrogenase complex DNA-binding transcriptional dual regulator	N	

Change in gene expression	Gene name	Drugs resisted	Gene function	Previously associated with drug resistance?	Notes
Overexpression	slyA	PHM	SlyA DNA-binding transcriptional activator	N	
Overexpression	yejG	PHM	predicted protein	N	
Overexpression	ylcG	PHM	DLP12 prophage; small membrane protein	N	
Overexpression	yrbL	PHM	predicted protein	N	
Overexpression	entS	RIF	enterobactin efflux transporter	Y	An entS insertion mutant has increased susceptibility to mitomycin C (Han et al, 2010).
Overexpression	gadE	RIF	'Glutamic acid decarboxylase' DNA-binding transcriptional activator	Y	gadE activates the glutamic acid decarboxylase (GAD) acid resistance system, and multi-drug efflux genes (Tucker et al, 2003).
Overexpression	gfcC	RIF	conserved protein	N	
Overexpression	nuoI	RIF	NADH:ubiquinone oxidoreductase, chain I	N	
Overexpression	cynS	SCM	cyanase	N	cynS may function in detoxification of cyanate (Anderson et al, 1990).
Overexpression	nudB	SCM, SMX	dihydroneopterin triphosphate pyrophosphohydrolase	N	nudB catalyzes the first committed step in the synthesis of folate (Suzuki & Brown, 1974).
Overexpression	pyrG	SCM, SMX	CTP synthetase	N	
Overexpression	ydiZ	SCM	predicted protein	N	
Overexpression	ykfF	SCM	predicted protein	N	
Overexpression	ymgG	SCM	predicted protein	N	
Overexpression	dksA	SMX	RNA polymerase-binding transcription factor	N	While dksA overexpression resists SMX, dksA deletion resists RIF
Overexpression	puuP	SPX	proton dependent putrescine transporter	N	

Change in gene expression	Gene name	Drugs resisted	Gene function	Previously associated with drug resistance?	Notes
Overexpression	fabI	TCL	enoyl acyl carrier protein reductase	Y	fabI is the target of Triclosan (Heath et al, 1998).
Overexpression	folA	TMP	dihydrofolate reductase	Y	folA is the target of Trimethoprim (Miovic & Pizer, 1971).
Overexpression	folM	TMP	dihydromonapterin reductase / dihydrofolate reductase	Y	folM is a dihydromonapterin reductase with weak activity as a dihydrofolate reductase (Giladi et al, 2003). While folM over-expression confers trimethoprim resistance, folM deletion confers sulfonamide resistance.
Overexpression	creA	VAN	conserved protein	N	
Deletion	ddpF	AMK	putative ATP-binding component of an ABC transporter	N	While ddpF overexpression resists CFS, CLX, MEC, PHD, ddpF deletion resists AMK
Deletion	gnsA	AMK	predicted regulator of phosphatidylethanolamine synthesis	N	
Deletion	pheM	AMK	phenylalanyl-tRNA synthetase operon leader peptide	N	
Deletion	ydjI	AMK	predicted aldolase	N	
Deletion	sbmA	BLM	peptide antibiotic transporter	Y	Loss of sbmA function confers resistance to proline-rich antimicrobial peptides (Mattiuzzo et al, 2007).
Deletion	asmA	CFS	predicted outer membrane protein assembly protein	N	asmA is required for assembly of the porins through which cephalosporins enter the cell (Misra & Miao, 1995).
Deletion	hrpB	CFS	predicted ATP-dependent helicase	N	
Deletion	yceG	CFS, VAN	predicted aminodeoxychorismate lyase	N	
Deletion	cadB	CLI	lysine:cadaverine antiporter	N	cadB is part of the lysine-dependent acid resistance system 4 (Meng & Bennett, 1992).
Deletion	rpmG	CLI	50S ribosomal subunit protein L33	N	

Change in gene expression	Gene name	Drugs resisted	Gene function	Previously associated with drug resistance?	Notes
Deletion	speA	CLI	biosynthetic arginine decarboxylase	N	speA catalyzes the first step in putrescine biosynthesis (Wu & Morris, 1973).
Deletion	speB	CLI	agmatinase	N	speB catalyzes the second step in putrescine biosynthesis (Satishchandran & Boyle, 1986).
Deletion	lon	CLX	DNA-binding, ATP-dependent protease	Y	Loss of lon function stabilizes marA to confer antibiotic resistance (Nicoloff et al, 2006).
Deletion	ompF	CLX, FOX	outer membrane porin F	Y	ompF is the primary route of cell entry for many beta-lactams, particularly cephalosporins (Nikaido, 1989).
Deletion	ompR	CLX, FOX	OmpR response regulator	Y	ompR is the transcriptional activator of ompF; loss of ompR prevents synthesis of ompF (Tsui et al, 1988).
Deletion	atpB	CRB	ATP synthase F0 complex - a subunit	N	
Deletion	atpE	CRB	ATP synthase F0 complex - c subunit	N	
Deletion	cpxA	CRB	CpxA sensory histidine kinase	N	cpxA is part of the stress response pathway to cell envelope damage (Pogliano et al, 1997).
Deletion	rnhA	ERY	RNase HI	N	
Deletion	sulA	ERY	SOS cell division inhibitor	N	
Deletion	ycbZ	ERY, SPR	putative ATP-dependent protease	N	
Deletion	fepB	MEC	ferric enterobactin ABC transporter	N	
Deletion	fepC	MEC	ferric enterobactin ABC transporter	N	
Deletion	fepG	MEC	ferric enterobactin ABC transporter	N	
Deletion	fes	MEC	enterochelin esterase	N	fes hydrolyzes ferric enterobactin (Langman et al, 1972).

Change in gene expression	Gene name	Drugs resisted	Gene function	Previously associated with drug resistance?	Notes
Deletion	glnD	MEC	uridylyltransferase	N	
Deletion	pdxA	MEC	4-hydroxy-L-threonine phosphate dehydrogenase, NAD-dependent	N	pdxA is required for pyridoxal phosphate synthesis (Lam et al, 1992).
Deletion	rodZ	MEC	transmembrane component of cytoskeleton	N	rodZ interacts with the target of mecillinam (mrdA) through the MreB cytoskeleton (Bendezu et al, 2009).
Deletion	ybjI	MEC	FMN phosphatase	N	ybjI possesses phosphatase activity against pyridoxal phosphate (Kuznetsova et al, 2006).
Deletion	dbpA	NIT	ATP-dependent RNA helicase, specific for 23S rRNA	N	
Deletion	lpcA	NIT	D-sedoheptulose 7-phosphate isomerase	N	lpcA catalyzes the first step in the synthesis of a core component of lipopolysaccharide; lpcA deletion confers sensitivity to some antibiotics by increasing cell permeability (Tamaki et al, 1971).
Deletion	nfsA	NIT	NADPH nitroreductase	Y	nfsA is required to activate nitrofurantoin to toxic reactive species, and so nfsA deletion confers nitrofurantoin resistance (McCalla et al, 1978).
Deletion	pstC	NIT	phosphate ABC transporter - membrane subunit	N	
Deletion	rfaC	NIT	ADP-heptose:LPS heptosyltransferase I	N	rfaC transfers heptose onto lipopolysaccharide (Kadrmas & Raetz, 1998)
Deletion	rfaD	NIT	ADP-L-glycero-D-mannoheptose-6-epimerase	N	rfaD catalyzes a step in the synthesis of lipopolysaccharide (Kneidinger et al, 2002).
Deletion	sspA	NIT	stringent starvation protein A	N	
Deletion	ybjC	NIT	predicted inner membrane protein	N	ybjC is co-transcribed with nfsA (Paterson et al, 2002). Polar effects on nfsA are a likely mechanism of resistance to nitrofurantoin.
Deletion	ratA	PHM	toxin of a predicted toxin-antitoxin pair	N	
Deletion	ubiF	PHM	2-octaprenyl-3-methyl-6-methoxy-1,4-benzoquinone hydroxylase	Y	uniF catalyzes a step in the ubiquinone biosynthesis pathway. Previously identified as a phleomycin resistant mutant (Collis & Grigg, 1989).

**Change in gene expression**	**Gene name**	**Drugs resisted**	**Gene function**	**Previously associated with drug resistance?**	**Notes**
Deletion	ubiG	PHM	bifunctional 3-demethylubiquinone-*8* 3-*O*-methyltransferase and 2-octaprenyl-6-hydroxyphenol methylase	Y	ubiG catalyzes a step in the ubiquinone biosynthesis pathway. ubiG deletion produces a similar defect to a Phleomycin-resistant mutation in ubiF
Deletion	ubiH	PHM	2-octaprenyl-6-methoxyphenol hydroxylase	Y	ubiH catalyzes a step in the ubiquinone biosynthesis pathway. ubiH deletion produces a similar defect to a Phleomycin-resistant mutation in ubiF
Deletion	ycgB	PHM	conserved protein	N	
Deletion	ymgG	PHM	predicted protein	N	
Deletion	dksA	RIF	RNA polymerase-binding transcription factor DksA	N	While dksA overexpression resists SMX, dksA deletion resists RIF
Deletion	marC	RIF	predicted transporter	N	
Deletion	ptsN	RIF	phosphotransferase system enzyme IIA, regulation of potassium transport	N	
Deletion	rlmL	RIF	fused dual 23S rRNA methyltransferase	N	
Deletion	yfgH	RIF	predicted outer membrane lipoprotein	N	
Deletion	ccmH	SCM, SMX	cytochrome c biogenesis protein	N	
Deletion	folM	SCM, SMX	dihydromonapterin reductase / dihydrofolate reductase	Y	While folM over-expression confers trimethoprim resistance, folM deletion confers sulfonamide resistance. A folM deletion has been previously observed to confer sulfonamide resistance (Girgis et al, 2009).
Deletion	folX	SCM, SMX	dihydroneopterin triphosphate 2'-epimerase	Y	A folX deletion has been previously observed to confer sulfonamide resistance (Girgis et al, 2009).
Deletion	ybiP	SPR	predicted hydrolase, inner membrane	N	

Change in gene expression	Gene name	Drugs resisted	Gene function	Previously associated with drug resistance?	Notes
Deletion	ygeO	SPR	predicted protein	N	ygeO is involved in the production of Extracellular Death Factor (Kolodkin-Gal et al, 2007).
Deletion	ymfJ	SPR	predicted protein	N	
Deletion	rsxC	TMP	member of SoxR-reducing complex	Y	rsxC deletion produces constitutive transcription of sox operon (Koo et al, 2003).
Deletion	dacA	VAN	D-alanyl-D-alanine carboxypeptidase IA (PBP5)	N	dacA is involved in production of the cellular target of vancomycin binding (D-alanyl-D-alanine). dacA deletion confers increased beta-lactam susceptibility (Sarkar et al, 2010).
Deletion	ycgB	PHM	conserved protein	N	

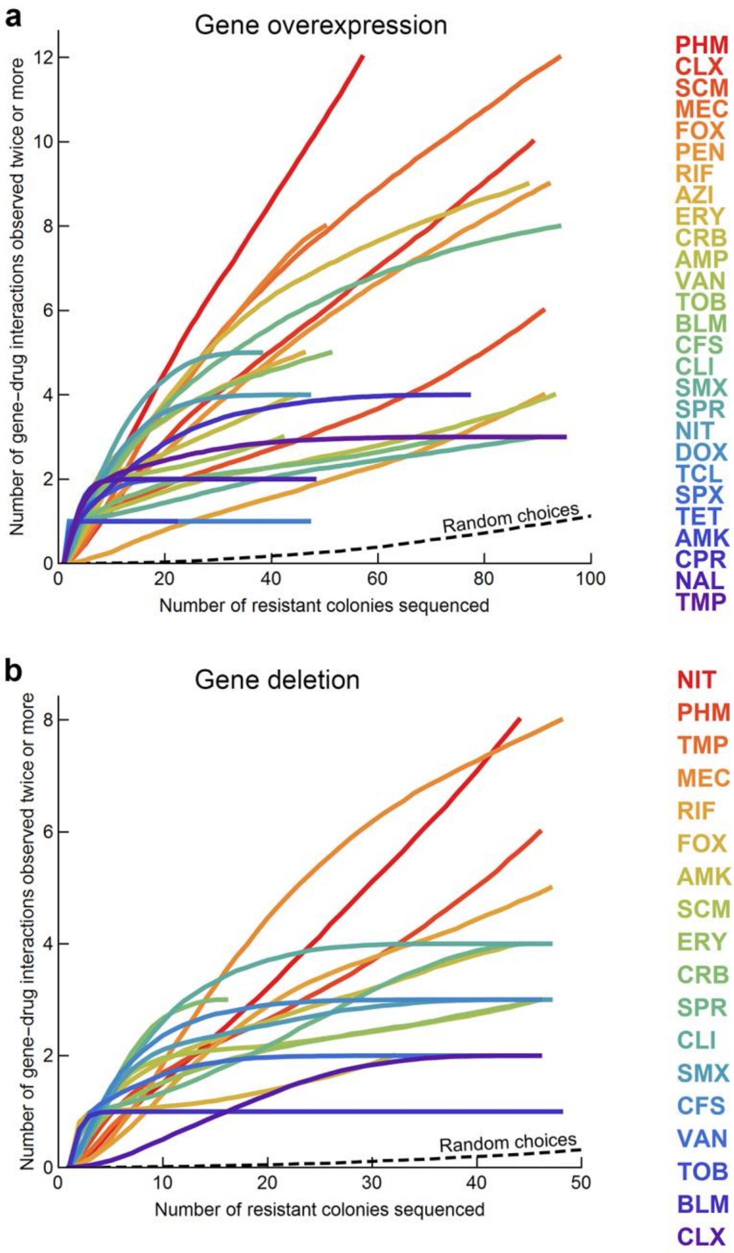

Figure S3. The extent of non-optimal gene expression is underestimated for some antibiotics. A statistical resampling approach was used to determine the average number of gene-drug interactions observed twice or more (the criteria for a 'hit') for each number of resistant colonies sequenced. The genes with altered expression (**a**, over-expressed, or **b**, deleted) from each sequenced colony were randomly ordered, and the number that were identified twice or more up to each position in the sequence was determined. This randomization process was repeated 1000 times for each combination of antibiotic and type of expression change and then averaged. This analysis revealed that for most antibiotics the assay is close to or has reached saturation, but for some antibiotics the extent of non-optimal gene expression is likely to be even greater than was revealed by the scale of this screen. In the legend of each plot (one each for overexpression and deletion screens), each antibiotic is ordered and colored by the degree of saturation of the resampling curve (specifically, the slope over last 5 colonies), such that antibiotics colored blue are those where the screen for non-optimal expression has reached saturation, and antibiotics colored red appear to be the farthest from saturation. A dashed black line indicates the number of genes that would be expected to be observed repeatedly if all choices were entirely random (average of 1000 sets of random selections out of the number of mutants in each library). In practice the false discovery rates for most antibiotics are lower than is indicated by this line because many gene expression changes are observed thrice or more and are undoubtedly not random choices (see SI Note). Note that resampling curves for the overexpression mutants sometimes halt in the vicinity of 48; for these cases, resistant colonies were observed only with high IPTG or only with low IPTG (up to 48 colonies were sequenced in each condition).

Table S2. Mechanisms of drug resistance mediated by changes in gene expression.

Genetic change	Putative resistance mechanism
**Drug efflux**	
marA, soxS	Transcriptional activation of multidrug resistance systems including efflux pumps
Δlon, ΔrsxC	Loss of enzyme required for inactivation of *mar* or *sox* systems, respectively
ycjR	Component of SdsRQP efflux pump
baeR	Increased transcription of MdtABC efflux pump
**Modify target process**	
sbmC	Inhibits DNA Gyrase and confers resistance to DNA damage by phleomycin
ΔdacA	Alters the peptidoglycan moiety bound by vancomycin
ΔrodZ	Loss of a binding partner of the target of mecillinam confers mecillinam resistance
ΔdksA	Loss of an RNA Polymerase binding protein increases resistance to the RNA Polymerase inhibitor rifamycin SV
hflX	Over-expression of ribosome component increases resistance to the translation inhibitors clindamycin and erythromycin
ΔrpmG	Loss of ribosome component increases clindamycin resistance
**Altered permeability**	
ΔompF, ΔompR, ΔasmA	Loss of the porins through which cephalosporins enter the cell
ΔsbmA	Loss of a transporter through which antimicrobial peptides enter the cell
amiA	Increased expression of a peptidoglycan amidase
bssR	Increased expression of a biofilm regulator
**Acid resistance systems**	
cadA, cadB	Activation of Lysine-dependent acid resistance system
gadE, gadW, ydeO	Activation of Glutamic acid decarboxylase acid resistance system
**Chemical modification of drug**	
ΔnfsA	Loss of enzyme that catalyzes pro-drug activation
ampC	Expression of enzyme that inactivates drug
**Increased flux through target pathway**	
nudB	Increased rate of first reaction in folic acid synthesis pathway
ΔfolM, ΔfolX	Increased flux through folic acid synthesis pathway by preventing substrate use for tetrahydromonapterin synthesis
folM	Drug-insensitive replacement for a drug-inhibited enzyme
folA, mrcB, mrdA, fabI	Increased expression of a drug-inhibited enzyme
**Lipopolysaccharide metabolism**	
ΔlpcA, ΔrfaC, ΔrfaD	Defects in lipopolysaccharide synthesis and modification
eptB	Increased phosphoethanolamine modification of lipopolysaccharide
**Polyamine metabolism and transport**	
puuP	Increased expression of putrescine transporter
rpmH	Decreased polyamine synthesis, but increased intracellular polyamines
ΔspeA, ΔspeB	Loss of putrescine biosynthesis
**Ubiquinone metabolism**	
ΔubiF, ΔubiG, ΔubiH	Loss of ubiquinone biosynthesis
nuoI	Increased expression of NADH:ubiquinone oxidoreductase
**Enterobactin transport and modification**	
ΔfepB, ΔfepC, ΔfepG	Loss of ferric enterobactin ABC transporter
Δfes	Loss of ferric enterobactin hydrolysis
entS	Increased expression of enterobactin transporter

Table S3. Many specific inhibitors are not resisted by overexpression of their target.
10 antibiotics in this screen specifically bind to one or two enzymes, yet only 4 of these were resisted by overexpression of their specific target gene(s). Conversely, antibiotic resistance can often be increased by the overexpression of particular non-target genes.

Antibiotic	Target overexpression confers resistance? Yes	No	Non-target genes that confer resistance when overexpressed (#)
Cephalexin		ftsI	12
Cefsulodin	mrcB	mrcA	3
Mecillinam	mrdA		11
Trimethoprim	folA		2
Sulfamethoxazole		folP	3
Sulfacetamide		folP	6
Ciprofloxacin		gyrA	4
Lomefloxacin		gyrA	0
Nalidixic acid		gyrA	2
Triclosan	fabI		0

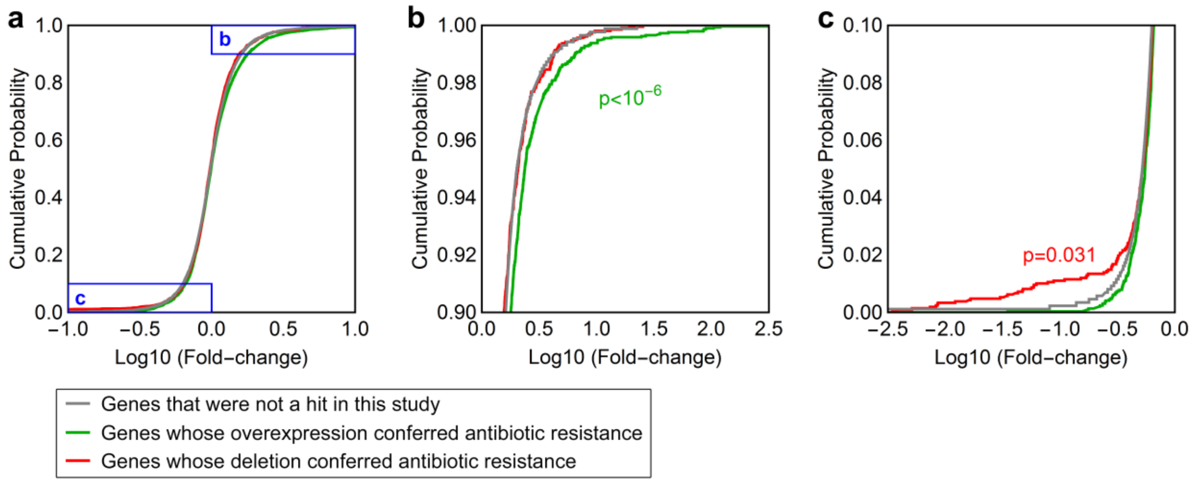

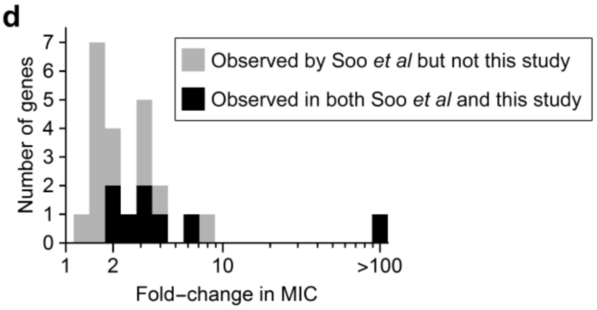

e

Comparison with Soo *et al.*	MIC < 2-fold	MIC ≥ 2-fold
Not observed in this study	9	7
Observed in this study	0	8

p = 0.009

f

Comparison with Hirakawa *et al.*	Single-stress resistance	Multi-stress resistance
Not observed in this study	10	2
Observed in this study	0	3

p = 0.02

Figure S4. Support for screen results from prior studies of antibiotic or stress resistance. a. Suzuki et al (2014) subjected 40 cultures of *E.coli* to directed evolution for antibiotic resistance (4 cultures for each of 10 antibiotics), and measured transcriptome-wide gene expression changes relative to the parent strain. Here, the cumulative distribution of these expression changes is plotted for three groups of genes: genes that were not a 'hit' in the non-optimality screen, genes whose overexpression conferred antibiotic resistance, and genes whose deletion conferred antibiotic resistance. The regions of strongest up-regulation and down-regulation are highlighted in blue and examined closer in panels **b** and **c**.
b. Examining the top 10% quantile of most up-regulated genes shows that 'hits' in this study for overexpression-mediated resistance are significantly more likely to be transcriptionally upregulated in drug-resistant *E.coli* ($p<10^{-6}$, Kolmogorov-Smirnov test). **c**. Examining the bottom 10% quantile of most down-regulated genes shows that 'hits' in this study for deletion-mediated resistance are significantly more likely to be transcriptionally down-regulated in drug-resistant *E.coli* (p=0.03, Kolmogorov-Smirnov test). **d**. Soo et al (2011) identified gene overexpression mutants with greatest resistance to a variety of toxins including some antibiotics, and measured changes in minimum inhibitory concentration (MIC) relative to a parent strain. Among antibiotics of similar class to those studied here, 24 cases of overexpression-mediated resistance were reported, of which 15 exhibited MIC change ≥ 2-fold. **e**. This study reproduced a significantly larger number of genes from Soo *et al* with MIC ≥ 2-fold (p=0.009, Fisher Exact Test). **f.** Hirakawa et al (2003) overexpressed response regulator genes in *E.coli* and measured which conferred resistant to individual or multiple chemical toxins (including only one antibiotic, erythromycin, present in this study). This study reproduced a significantly larger number of response regulator genes conferring multi-toxin resistance than single-toxin resistance (p=0.02, Fisher Exact Test).

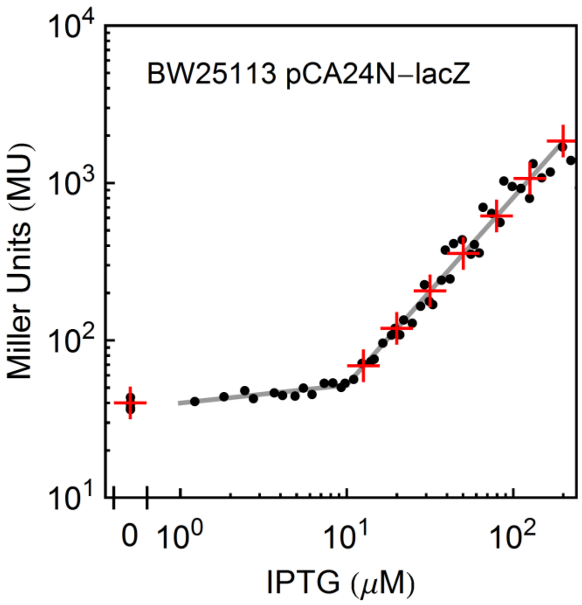

Figure S5. Measurement of IPTG-induced transcription of antibiotic resistance genes by beta-galactosidase assays. The pCA24N plasmid used to express Open Reading Frames in the ASKA library was engineered to express *lacZ*, and transformed into BW25113. Liquid cultures of this strain were prepared as for growth rate assays, across a gradient of IPTG concentrations. In early log phase, promoter activity was measured in Miller Units by a kinetic beta-galactosidase assay (black points). The resulting data is well described by two straight lines on a double-log plot (gray lines). From this data, eight IPTG concentrations were chosen for the growth rate assays in Figures 3 and 4, that produced evenly log-distributed amounts of promoter activity (in Miller Units) over a 50-fold dynamic range (red crosses). The growth rate measurements of Figures 3 and 4 were performed in parallel with and under identical conditions to those of (Palmer & Kishony, 2014), and so this same calibration between IPTG and MU is applied to both studies.

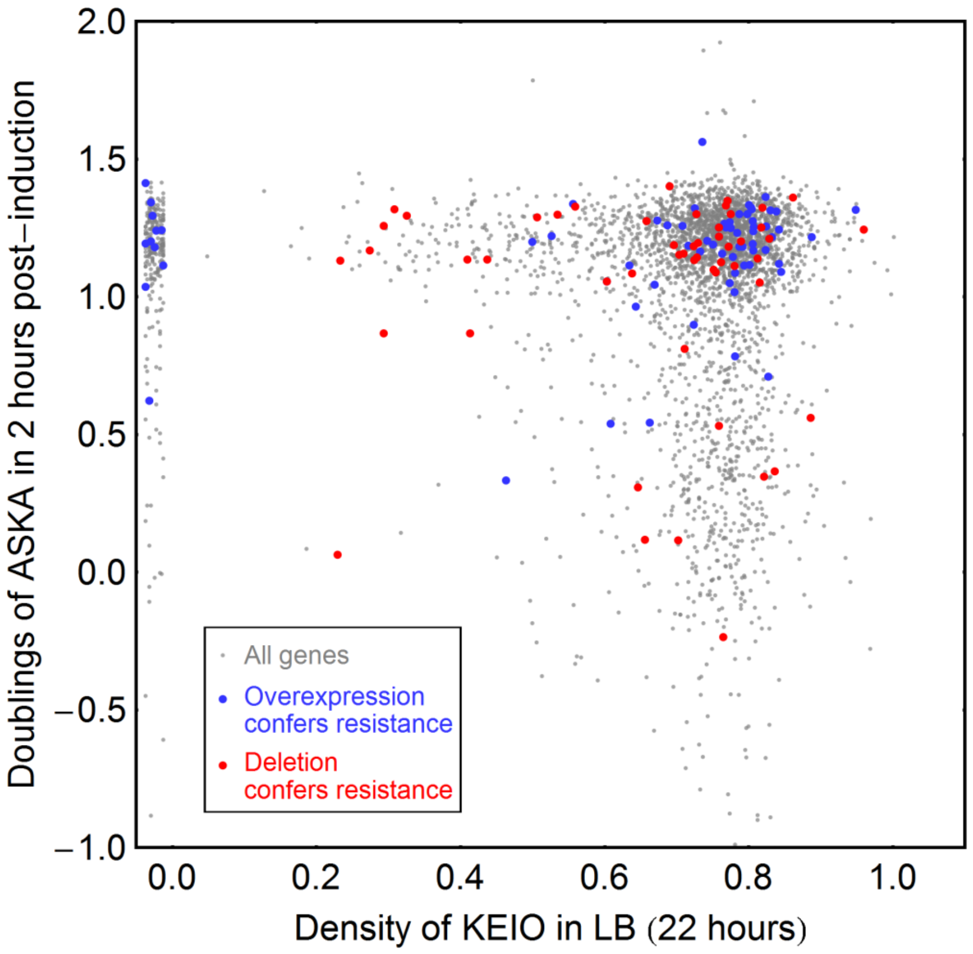

Figure S6. Most gene deletion and overexpression mutants in *Escherichia coli* have modest effects on fitness. Articles describing the creation of the *E. coli* deletion (Baba et al, 2006) and overexpression (Kitagawa et al, 2005) libraries also report measurements of the fitness effects of these mutations. For the deletion library, the optical density of each strain at 600nm was reported after 22 hours of growth in Lysogeny Broth (LB) at 37°C without shaking (horizontal axis) (Baba et al, 2006). For the overexpression library, each strain was grown in LB at 37°C to mid-log phase (OD600 = 0.5), IPTG was added to the media to final concentration 1mM (a very strong level of induction), and the culture was incubated for 2 hours. The number of doublings was calculated from OD600 readings before and after this 2 hour period of expression induction (vertical axis) (Kitagawa et al, 2005). Essential genes, where no deletion mutant can be created, are shown on the far left of the scatter plot. Overexpression mutants that confer resistance to some antibiotic in our screen (blue) rarely confer a significant growth defect when overexpressed (only 7 of 70 gene overexpression mutants have less than 80% of the genome-wide median number of doublings). Deletion mutants that confer resistance to some antibiotic in our screen (red) are somewhat more likely to confer a growth defect when deleted (14 of 52 gene deletion mutants have less than 80% of the genome-wide median density), but still the majority have only a modest effect.

Figure S7. Non-optimal use of antibiotic resistance genes under antibiotic stress. Microtiter plates containing 2-dimensional gradients of IPTG and antibiotic were inoculated with either a wildtype strain (WT = BW25113 pCA24N-ΔpT5lac-*yfp* pCSλ), a strain lacking a gene of interest (Δ*gene* = BW25113 *gene*::FRT pCA24N-ΔpT5lac-*yfp* pCSλ), or a strain with experimentally controlled expression of the gene of interest (BW25113 *gene*::FRT pCA24N-*gene* pCSλ). Plates were incubated at 30°C in a scintillation counter and growth rates were measured based on bioluminescence, generated by the pCSλ plasmid (Methods). **a**, The use of *marA* and *soxS* was measured in a panel of 9 antibiotics. **b**, The use of *ampC* was measured in ampicillin and cephalexin, and the use of *sbmC* was measured in phleomycin.

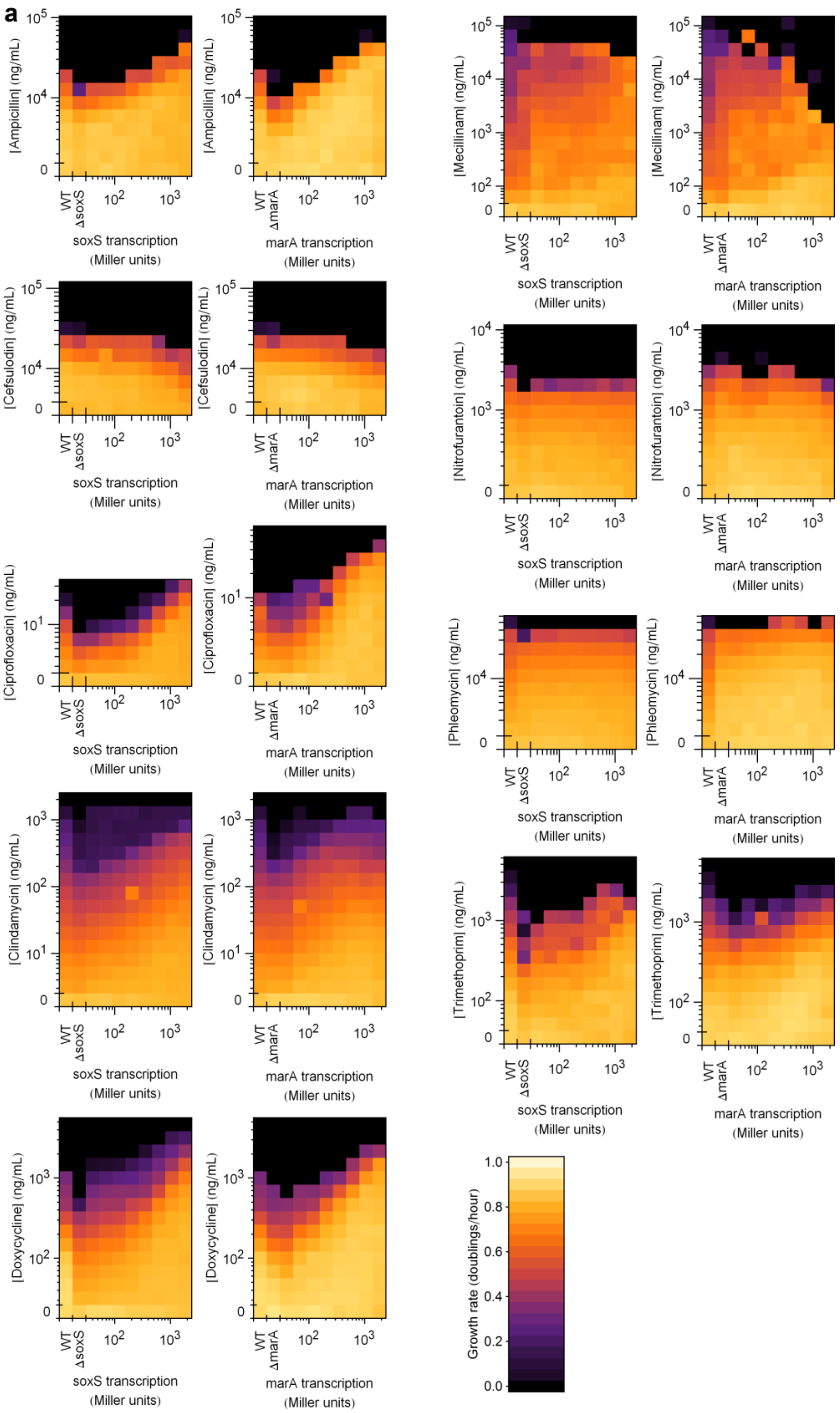

Figure S7 (continued).

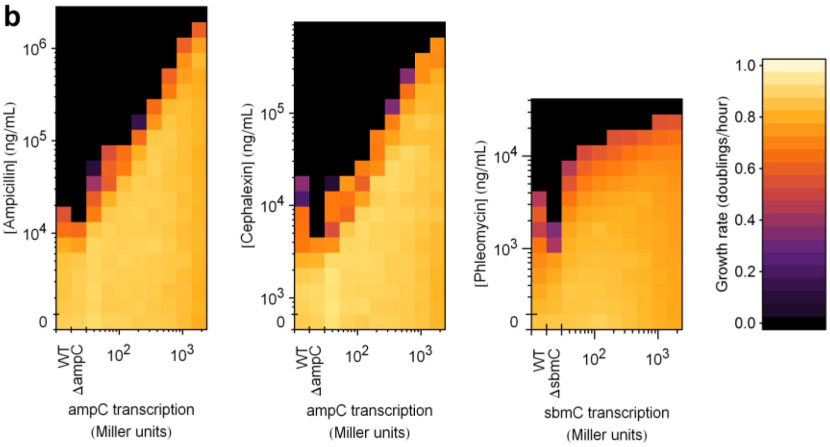

Figure S7 (continued).

Table S4. Concentrations and volumes of antibiotics in the pooled drug diffusion assay.

Abbrev.	Antibiotic	Solvent	Concentration (mg/mL)	Volume (µL)
AMK	Amikacin	water	50	20
AMP	Ampicillin	water	100	20
AZI	Azithromycin	water	10	20
BLM	Bleomycin	water	20	20
CFS	Cefsulodin	water	10	20
CLI	Clindamycin	water	50	20
CLX	Cephalexin	water	10	20
COL	Colistin	water	20	10
CPR	Ciprofloxacin	water	1	1
CRB	Carbenicillin	water	50	20
DOX	Doxycycline	ethanol	5	20
ERY	Erythromycin	water	50	20
FOX	Cefoxitin	water	50	20
LOM	Lomefloxacin	water	20	2
MEC	Mecillinam	water	10	20
NAL	Nalidixic acid	water	15	20
NIT	Nitrofurantoin	dimethyl formamide	10	20
PEN	Penicillin	water	100	20
PHM	Phleomycin	water	5	20
PMB	Polymyxin B	water	20	20
RIF	Rifamycin SV	methanol	10	20
SCM	Sulfacetamide	DMSO	200	3*
SMX	Sulfamethoxazole	DMSO	100	1*
SPR	Spiramycin	ethanol	50	20
SPX	Spectinomycin	water	50	20
STR	Streptomycin	water	50	20
TCL	Triclosan	ethanol	100	2
TET	Tetracycline	ethanol	5	20
TMP	Trimethoprim	DMSO	10	1
TOB	Tobramycin	water	20	20
VAN	Vancomycin	water	50	20

*Antibiotic only dissolved well into agar when the solution was added to agar still wet with 150microL of bacterial culture overlay (in all other cases antibiotic was added after culture overlay dried).

SI Note: False Discovery Rate of the screen for non-optimal gene expression.

A change in gene expression that is identified in *one* drug resistant colony may be the result of a spontaneous resistance mutation in that cell other than the artificial gene deletion or overexpression. Accordingly, gene-drug interactions are only called when the same change in gene expression is observed *two or more* independent times to confer resistance to the same drug. The probability of Type I error in this assay is thus the probability that a random colony would spontaneously appear twice or more, which can be calculated as an instance of the 'Birthday problem', which concerns the likelihood that the same birthday may be found twice amongst a group of people. Importantly, there are many more strains in the gene deletion and overexpression libraries than there are days in a year.

First, we establish by monte carlo simulation that a gene-drug interaction observed *three or more* times has a negligibly small probability of being false. For each drug, 48 colonies (if any were present) were picked and sequenced under each condition of deletion, mild overexpression, and strong overexpression. For the purpose of determining which genes are repeatedly identified, the gene list from mild and strong overexpression was merged, for a total of up to 96 colonies sequenced. If 48 strains were randomly picked from the 3985 gene deletion strains (Baba et al, 2006), the total probability that any would be randomly selected three or more times is 0.1%; with a probability per gene of 3×10^{-7} (by 10^6 simulated selections of 48 random strains out of 3985). Similarly, if 96 strains were randomly picked from the 4267 gene overexpression strains (Kitagawa et al, 2005), the total probability that any would be randomly selected three or more times is less than 0.8%, with a probability per gene of 2×10^{-6} (by 10^6 simulated selections of 96 random strains out of 4267). As most genes identified by sequencing were observed repeatedly, there were not as many as 48 or 96 *random* selections, and thus these values are vast overestimates.

Having established that only genes identified *exactly twice* warrant the quantification of a False Discovery Rate (FDR), we apply the solution to the birthday problem:
Probability that at least 2 of n people share a birthday = $(1 - 365! / (365^n \times (365 - n)!))$.
This formula is used to calculate the probability of a least a single type I error (false positive) for a particular drug and expression condition (deletion or overexpression) by replacing '365 days' with the number of distinct strains in a given mutant library, and replacing the number of people n with the number of genes identified once or twice. Having established that genes identified thrice or more are certainly genuine, these counts do not contribute to the number of random selections. The FDR is then the probability of a type I error divided by the total number of 'hits' called (genes identified twice or more) for that particular drug and expression condition (see Table below). In cases where all hits are observed three or more times (no genes are identified exactly twice), the FDR is approximately zero, as demonstrated by simulation. The mean and median FDR is 0.6% and 0.015%, respectively, with the sum total of type 1 error probabilities indicating that probably two gene-drug interactions are false across the data set of approximately 200 interactions. Because genes observed only once may in fact be true positives (that did not happen to be repeatedly identified) rather than random selections, these calculations are likely to overestimate FDR. Only overexpression mutants conferring Rifamycin resistance have a notably high FDR, of 13%: the genes concerned are *gadE* (a known activator of multi-drug efflux pumps), *gfcC*, and *nuoI*; (*entS* is of no concern on account of 9 independent observations).

Drug	Number of genes observed: once	twice	≥ 3 times	P(number of type 1 errors ≥1)	FDR of genes observed twice
**Overexpression**					
AMK	3	0	1	0	0
AMP	1	2	2	0.0007	0.0002
AZI	0	1	4	0	0
BLM	11	1	4	0.0154	0.0031
CFS	19	1	2	0.0436	0.0145
CLI	4	1	7	0.0023	0.0003
CLX	35	5	5	0.1675	0.0168
CPR	4	0	4	0	0
CRB	2	1	3	0.0007	0.0002
DOX	1	0	2	0	0
ERY	17	2	7	0.0393	0.0044
FOX	6	2	6	0.0065	0.0008
MEC	27	4	8	0.1035	0.0086
NAL	3	0	2	0	0
NIT	3	0	4	0	0
PEN	31	3	6	0.1235	0.0137
PHM	4	6	6	0.0105	0.0009
RIF	76	3	1	0.5164	0.1291
SCM	40	4	2	0.1995	0.0332
SMX	12	1	2	0.0181	0.0060
SPR	3	0	5	0	0
SPX	1	0	1	0	0
TCL	0	0	1	0	0
TET	1	0	2	0	0
TMP	0	0	3	0	0
TOB	1	0	2	0	0
VAN	7	1	2	0.0065	0.0022
**Deletion**					
AMK	5	1	3	0.0038	0.0009
BLM	8	0	1	0	0
CFS	3	0	3	0	0
CLI	4	0	4	0	0
CLX	37	0	2	0	0
CRB	2	0	3	0	0
ERY	14	1	2	0.0260	0.0087
FOX	1	1	1	0.0003	0.0001
MEC	13	2	6	0.0260	0.0033
NIT	13	5	3	0.0377	0.0047
PHM	11	4	2	0.0260	0.0043
RIF	24	2	3	0.0785	0.0157
SCM	9	1	2	0.0112	0.0037
SMX	8	0	3	0	0
SPR	3	0	4	0	0
TMP	5	0	1	0	0
VAN	1	0	2	0	0

The data presented in Figure 2 includes additionally some interactions where a specific change in gene expression was repeatedly observed to resist one drug, and was once observed to resist another drug of the same class (pale links in Figure 2a). For this set of interactions, monte carlo simulations were used to determine the probability of type 1 errors for the drugs concerned, that is, given the number of changes in gene expression that were observed only once, how likely is one to randomly match one of the changes in gene expression observed repeatedly in the same drug class. The mean FDR for this additional set of 14 gene-drug interactions was determined to be 5.0%. The single highest FDR was 13%, concerning the CLX-Δ*ompF* interaction, which is known to be true (Nikaido, 1989).

Supplementary References

Amabile-Cuevas CF, Demple B (1991) Molecular characterization of the soxRS genes of Escherichia coli: two genes control a superoxide stress regulon. *Nucleic acids research* **19:** 4479-4484

Anderson PM, Sung YC, Fuchs JA (1990) The cyanase operon and cyanate metabolism. *FEMS microbiology reviews* **7:** 247-252

Baba T, Ara T, Hasegawa M, Takai Y, Okumura Y, Baba M, Datsenko KA, Tomita M, Wanner BL, Mori H (2006) Construction of Escherichia coli K-12 in-frame, single-gene knockout mutants: the Keio collection. *Mol Syst Biol* **2:** 2006 0008

Baquero MR, Bouzon M, Varea J, Moreno F (1995) sbmC, a stationary-phase induced SOS Escherichia coli gene, whose product protects cells from the DNA replication inhibitor microcin B17. *Mol Microbiol* **18:** 301-311

Barth E, Gora KV, Gebendorfer KM, Settele F, Jakob U, Winter J (2009) Interplay of cellular cAMP levels, {sigma}S activity and oxidative stress resistance in Escherichia coli. *Microbiology* **155:** 1680-1689

Bendezu FO, Hale CA, Bernhardt TG, de Boer PA (2009) RodZ (YfgA) is required for proper assembly of the MreB actin cytoskeleton and cell shape in E. coli. *The EMBO journal* **28:** 193-204

Cohen SP, Hachler H, Levy SB (1993) Genetic and functional analysis of the multiple antibiotic resistance (mar) locus in Escherichia coli. *J Bacteriol* **175:** 1484-1492

Collis CM, Grigg GW (1989) An Escherichia coli mutant resistant to phleomycin, bleomycin, and heat inactivation is defective in ubiquinone synthesis. *J Bacteriol* **171:** 4792-4798

Dinh T, Paulsen IT, Saier MH, Jr. (1994) A family of extracytoplasmic proteins that allow transport of large molecules across the outer membranes of gram-negative bacteria. *J Bacteriol* **176:** 3825-3831

Giladi M, Altman-Price N, Levin I, Levy L, Mevarech M (2003) FolM, a new chromosomally encoded dihydrofolate reductase in Escherichia coli. *J Bacteriol* **185:** 7015-7018

Girgis HS, Hottes AK, Tavazoie S (2009) Genetic architecture of intrinsic antibiotic susceptibility. *PLoS One* **4:** e5629

Han X, Dorsey-Oresto A, Malik M, Wang JY, Drlica K, Zhao X, Lu T (2010) Escherichia coli genes that reduce the lethal effects of stress. *BMC microbiology* **10:** 35

Heath RJ, Yu YT, Shapiro MA, Olson E, Rock CO (1998) Broad spectrum antimicrobial biocides target the FabI component of fatty acid synthesis. *The Journal of biological chemistry* **273:** 30316-30320

Hirakawa H, Nishino K, Hirata T, Yamaguchi A (2003) Comprehensive studies of drug resistance mediated by overexpression of response regulators of two-component signal transduction systems in Escherichia coli. *J Bacteriol* **185:** 1851-1856

Kadrmas JL, Raetz CR (1998) Enzymatic synthesis of lipopolysaccharide in Escherichia coli. Purification and properties of heptosyltransferase i. *The Journal of biological chemistry* **273:** 2799-2807

Keseler IM, Collado-Vides J, Santos-Zavaleta A, Peralta-Gil M, Gama-Castro S, Muniz-Rascado L, Bonavides-Martinez C, Paley S, Krummenacker M, Altman T, Kaipa P, Spaulding A, Pacheco J, Latendresse M, Fulcher C, Sarker M, Shearer AG, Mackie A, Paulsen I, Gunsalus RP et al (2011) EcoCyc: a comprehensive database of Escherichia coli biology. *Nucleic acids research* **39:** D583-590

Kim I, Kim J, Min B, Lee C, Park C (2007) Screening of genes related to methylglyoxal susceptibility. *J Microbiol* **45:** 339-343

Kitagawa M, Ara T, Arifuzzaman M, Ioka-Nakamichi T, Inamoto E, Toyonaga H, Mori H (2005) Complete set of ORF clones of Escherichia coli ASKA library (a complete set of E. coli K-12 ORF archive): unique resources for biological research. *DNA Res* **12:** 291-299

Kneidinger B, Marolda C, Graninger M, Zamyatina A, McArthur F, Kosma P, Valvano MA, Messner P (2002) Biosynthesis pathway of ADP-L-glycero-beta-D-manno-heptose in Escherichia coli. *J Bacteriol* **184:** 363-369

Kolodkin-Gal I, Hazan R, Gaathon A, Carmeli S, Engelberg-Kulka H (2007) A linear pentapeptide is a quorum-sensing factor required for mazEF-mediated cell death in Escherichia coli. *Science* **318:** 652-655

Kong KF, Schneper L, Mathee K (2010) Beta-lactam antibiotics: from antibiosis to resistance and bacteriology. *APMIS : acta pathologica, microbiologica, et immunologica Scandinavica* **118:** 1-36

Koo MS, Lee JH, Rah SY, Yeo WS, Lee JW, Lee KL, Koh YS, Kang SO, Roe JH (2003) A reducing system of the superoxide sensor SoxR in Escherichia coli. *The EMBO journal* **22:** 2614-2622

Kuznetsova E, Proudfoot M, Gonzalez CF, Brown G, Omelchenko MV, Borozan I, Carmel L, Wolf YI, Mori H, Savchenko AV, Arrowsmith CH, Koonin EV, Edwards AM, Yakunin AF (2006) Genome-wide analysis of substrate specificities of the Escherichia coli haloacid dehalogenase-like phosphatase family. *The Journal of biological chemistry* **281:** 36149-36161

Lam HM, Tancula E, Dempsey WB, Winkler ME (1992) Suppression of insertions in the complex pdxJ operon of Escherichia coli K-12 by lon and other mutations. *J Bacteriol* **174:** 1554-1567

Langman L, Young IG, Frost GE, Rosenberg H, Gibson F (1972) Enterochelin system of iron transport in Escherichia coli: mutations affecting ferric-enterochelin esterase. *J Bacteriol* **112:** 1142-1149

Linstrom EB, Boman HG, Steele BB (1970) Resistance of Escherichia coli to penicillins. VI. Purification and characterization of the chromosomally mediated penicillinase present in ampA-containing strains. *J Bacteriol* **101:** 218-231

Masuda N, Church GM (2003) Regulatory network of acid resistance genes in Escherichia coli. *Mol Microbiol* **48:** 699-712

Mattiuzzo M, Bandiera A, Gennaro R, Benincasa M, Pacor S, Antcheva N, Scocchi M (2007) Role of the Escherichia coli SbmA in the antimicrobial activity of proline-rich peptides. *Mol Microbiol* **66:** 151-163

McCalla DR, Kaiser C, Green MH (1978) Genetics of nitrofurazone resistance in Escherichia coli. *J Bacteriol* **133:** 10-16

Meng SY, Bennett GN (1992) Nucleotide sequence of the Escherichia coli cad operon: a system for neutralization of low extracellular pH. *J Bacteriol* **174:** 2659-2669

Miovic M, Pizer LI (1971) Effect of trimethoprim on macromolecular synthesis in Escherichia coli. *J Bacteriol* **106:** 856-862

Misra R, Miao Y (1995) Molecular analysis of asmA, a locus identified as the suppressor of OmpF assembly mutants of Escherichia coli K-12. *Mol Microbiol* **16:** 779-788

Nagakubo S, Nishino K, Hirata T, Yamaguchi A (2002) The putative response regulator BaeR stimulates multidrug resistance of Escherichia coli via a novel multidrug exporter system, MdtABC. *J Bacteriol* **184:** 4161-4167

Nicoloff H, Perreten V, McMurry LM, Levy SB (2006) Role for tandem duplication and lon protease in AcrAB-TolC- dependent multiple antibiotic resistance (Mar) in an Escherichia coli mutant without mutations in marRAB or acrRAB. *J Bacteriol* **188:** 4413-4423

Nikaido H (1989) Outer membrane barrier as a mechanism of antimicrobial resistance. *Antimicrob Agents Chemother* **33:** 1831-1836

Palmer AC, Kishony R (2014) Opposing effects of target overexpression reveal drug mechanisms. *Nature communications* **5:** 4296

Panagiotidis CA, Huang SC, Canellakis ES (1995) Relationship of the expression of the S20 and L34 ribosomal proteins to polyamine biosynthesis in Escherichia coli. *The international journal of biochemistry & cell biology* **27:** 157-168

Paterson ES, Boucher SE, Lambert IB (2002) Regulation of the nfsA Gene in Escherichia coli by SoxS. *J Bacteriol* **184:** 51-58

Pogliano J, Lynch AS, Belin D, Lin EC, Beckwith J (1997) Regulation of Escherichia coli cell envelope proteins involved in protein folding and degradation by the Cpx two-component system. *Genes & development* **11:** 1169-1182

Polissi A, De Laurentis W, Zangrossi S, Briani F, Longhi V, Pesole G, Deho G (2003) Changes in Escherichia coli transcriptome during acclimatization at low temperature. *Research in microbiology* **154:** 573-580

Reynolds CM, Kalb SR, Cotter RJ, Raetz CR (2005) A phosphoethanolamine transferase specific for the outer 3-deoxy-D-manno-octulosonic acid residue of Escherichia coli lipopolysaccharide. Identification of the eptB gene and Ca2+ hypersensitivity of an eptB deletion mutant. *The Journal of biological chemistry* **280:** 21202-21211

Sarkar SK, Chowdhury C, Ghosh AS (2010) Deletion of penicillin-binding protein 5 (PBP5) sensitises Escherichia coli cells to beta-lactam agents. *International journal of antimicrobial agents* **35:** 244-249

Satishchandran C, Boyle SM (1986) Purification and properties of agmatine ureohydrolyase, a putrescine biosynthetic enzyme in Escherichia coli. *J Bacteriol* **165:** 843-848

Soo VW, Hanson-Manful P, Patrick WM (2011) Artificial gene amplification reveals an abundance of promiscuous resistance determinants in Escherichia coli. *Proc Natl Acad Sci U S A* **108:** 1484-1489

Sprenger GA, Schorken U, Wiegert T, Grolle S, de Graaf AA, Taylor SV, Begley TP, Bringer-Meyer S, Sahm H (1997) Identification of a thiamin-dependent synthase in Escherichia coli required for the formation of the 1-deoxy-D-xylulose 5-phosphate precursor to isoprenoids, thiamin, and pyridoxol. *Proc Natl Acad Sci U S A* **94:** 12857-12862

Suzuki S, Horinouchi T, Furusawa C (2014) Prediction of antibiotic resistance by gene expression profiles. *Nature communications* **5:** 5792

Suzuki Y, Brown GM (1974) The biosynthesis of folic acid. XII. Purification and properties of dihydroneopterin triphosphate pyrophosphohydrolase. *The Journal of biological chemistry* **249:** 2405-2410

Takayama M, Ohyama T, Igarashi K, Kobayashi H (1994) Escherichia coli cad operon functions as a supplier of carbon dioxide. *Mol Microbiol* **11:** 913-918

Tamaki S, Sato T, Matsuhashi M (1971) Role of lipopolysaccharides in antibiotic resistance and bacteriophage adsorption of Escherichia coli K-12. *J Bacteriol* **105:** 968-975

Tsui P, Helu V, Freundlich M (1988) Altered osmoregulation of ompF in integration host factor mutants of Escherichia coli. *J Bacteriol* **170:** 4950-4953

Tucker DL, Tucker N, Ma Z, Foster JW, Miranda RL, Cohen PS, Conway T (2003) Genes of the GadX-GadW regulon in Escherichia coli. *J Bacteriol* **185:** 3190-3201

Vimr ER, Troy FA (1985) Identification of an inducible catabolic system for sialic acids (nan) in Escherichia coli. *J Bacteriol* **164:** 845-853

Wei Y, Vollmer AC, LaRossa RA (2001) In vivo titration of mitomycin C action by four Escherichia coli genomic regions on multicopy plasmids. *J Bacteriol* **183:** 2259-2264

Wu WH, Morris DR (1973) Biosynthetic arginine decarboxylase from Escherichia coli. Purification and properties. *The Journal of biological chemistry* **248:** 1687-1695
