# Supplementary material for: Nonoptimal Gene Expression Creates Latent Potential for Antibiotic Resistance
Source: Mol Biol Evol. 2018 Aug 28;35(11):2669–84. doi: 10.1093/molbev/msy163 (PMC6231494; doi:10.1093/molbev/msy163)
Supplement: Supplementary Data [file msy163_supp.zip › msy163_Supp/Supplemental code.pdf]

Supporting code

Written for Wolfram Mathematica v11.0

Model of antibiotic perturbation of fitness-expression functions

Fitness as a function of drug concentration = f_a

In this model, this function does not need to follow a specific mathematical form, but should follow an experimentally observed dose-response to antibiotic treatment in a ‘wildtype’ bacterial strain (that is, in the absence of any experimental perturbation of gene expression).

Here as a simple illustration we will use $f_a = 1 - a$

(incidentally, this very simple form does provide a reasonable fit to much empirical dose-response data, provided with some scaling factor K so that $f_a = 1 - a/K$)

```
In[1]:= fa[a_] := 1 - a

(* custom axis specifications for log-scale *)
logframeticks = Join[Table[{x, "10"^ToString[x], {0.02, 0}}, {x, -10, 10}],
   Flatten[Table[Table[{Log[10, x], "", {0.01, 0}}, {x, 1 * 10^y, 9 * 10^y, 1 * 10^y}], {y, -10, 10, 1}], 1]];

Plot[fa[10^loga], {loga, -2, 0.2}, PlotRange → {0, 1}, AxesOrigin → {-2, 0},
 Frame → {{True, False}, {True, False}}, FrameTicks → {logframeticks, Automatic}, PlotRangePadding → None,
 BaseStyle → {FontFamily → "Arial", FontSize → 12}, FrameStyle → Directive[Black, Thickness[Medium]],
 FrameLabel → {"Antibiotic concentration\n(arbitrary units; MIC = 1)", "Relative Fitness"},
 PlotStyle → Directive[ColorData[3, 4], AbsoluteThickness[2]]]
```

Out[3]=

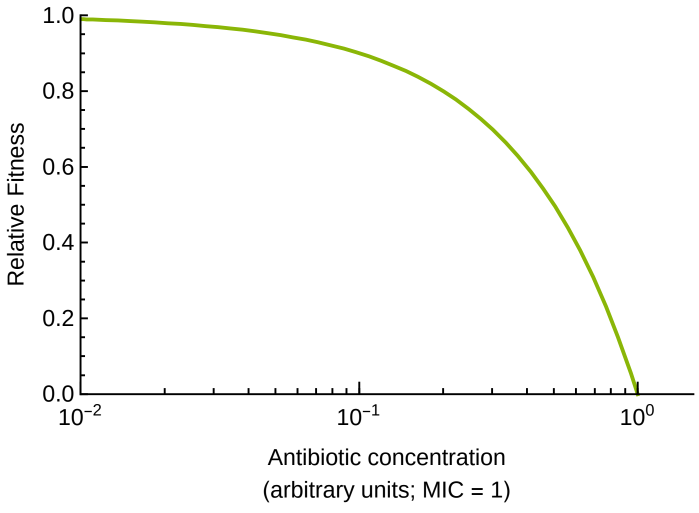

Fitness as a function of gene expression = f_g

In this model, this function does not need to follow a specific mathematical form, but should follow an experimentally observed fitness-expression function, that is, how fitness depends on the level of gene expression - in the absence of any antibiotic treatment.

Here as a simple illustration we will use a quadratic $f_g = 1 - \frac{(g-1)^2}{10}$, which captures the qualitative features of many fitness-expression functions: a modest fitness cost for gene deletion, and a gradually increasing fitness cost for overexpression, with some intermediate level of expression conferring maximum fitness.

In[4]:=
```
fg[g_] := 1 - (g - 1)^2 / 10

Plot[fg[g], {g, 0, 2}, PlotRange → {0, 1}, AxesOrigin → {0, 0}, Frame → {{True, False}, {True, False}}, FrameTicks → {Automatic, Automatic},
 PlotRangePadding → None, BaseStyle → {FontFamily → "Arial", FontSize → 12}, FrameStyle → Directive[Black, Thickness[Medium]],
 FrameLabel → {"Gene expression\n(arbitrary units; optimal expression = 1)", "Relative Fitness"},
 PlotStyle → Directive[Black, AbsoluteThickness[2]]]
```

Out[5]=

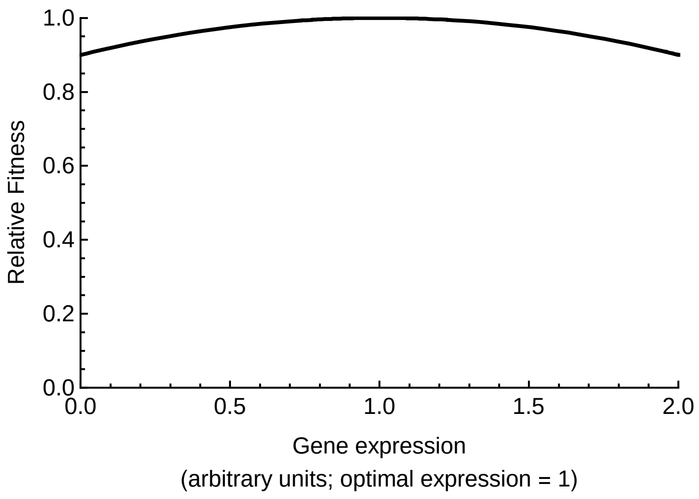

In the absence of any interaction between gene expression and drug potency, Fitness is the product of the independent effects of gene expression and antibiotic treatment on relative fitness:

Fitness $= f_g(g) \times f_a(a)$

In[6]:=
```
Fitness[a_, g_] := fg[g] * fa[a]
```

In[7]:=
```
(* a gradient of antibiotic concentrations *)
AntibioticConcentrations = Prepend[Table[10^loga, {loga, -1.75, 1.25, 0.25}], 0]
```

Out[7]=
```
{0, 0.0177828, 0.0316228, 0.0562341, 0.1, 0.177828, 0.316228, 0.562341, 1., 1.77828, 3.16228, 5.62341, 10., 17.7828}
```

In[8]:=
```
(* a list of plot colors: Black = no antibiotic treatment; yellow through magenta = increasing antibiotic doses *)
plotcolors = Join[{Black}, Table[Hue[i], {i, 0.15, 0, -0.03}], Table[Hue[i], {i, 1, 0.8, -0.03}]]
```

Out[8]=

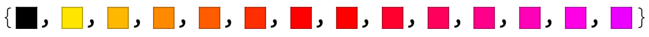

In[9]:=
```
ArrayPlot[Append[Reverse[Table[{x, x}, {x, 1, 14, 1}]], {0, 0}],
 ColorRules → Table[i → plotcolors[[i]], {i, 1, 14, 1}], ColorFunctionScaling → False, PlotRangePadding → None,
 FrameStyle → Directive[Black, Opacity[0]], Frame → {{True, False}, {False, False}}, FrameTicks →
  {Table[{i[[1]], Style[i[[2]], Opacity[1]], {0, 0}}, {i, {{2, "10"}, {4, "3"}, {6, "1"}, {8, "0.3"}, {10, "0.1"}, {12, "0.03"}, {14, "0"}}}],
   None}, BaseStyle → {FontFamily → "Arial", FontSize → 12}, ImageSize → {{500}, {250}},
 FrameLabel → {Style["[Antibiotic]  (arbitrary units)", Opacity[1]], None}, ImagePadding → {{55, 10}, {10, 10}}, AspectRatio → 10]
```

Out[9]=

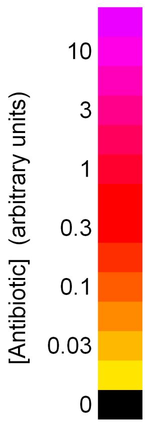

In this scenario, the fitness expression function is uniformly depressed by antibiotic treatment, with no change in the optimal level of gene expression:

In[10]:=
```
Show[Table[Plot[Fitness[AntibioticConcentrations[[i]], g], {g, 0, 2}, PlotRange → {0, 1}, PlotStyle → plotcolors[[i]]], {i, 1, 14, 1}],
 AxesOrigin → {0, 0}, Frame → {{True, False}, {True, False}}, FrameTicks → {Automatic, Automatic}, PlotRangePadding → None,
 BaseStyle → {FontFamily → "Arial", FontSize → 12}, FrameStyle → Directive[Black, Thickness[Medium]],
 FrameLabel → {"Gene expression\n(arbitrary units; optimal expression = 1)", "Relative Fitness"}, PlotStyle → AbsoluteThickness[2]]
```

Out[10]=

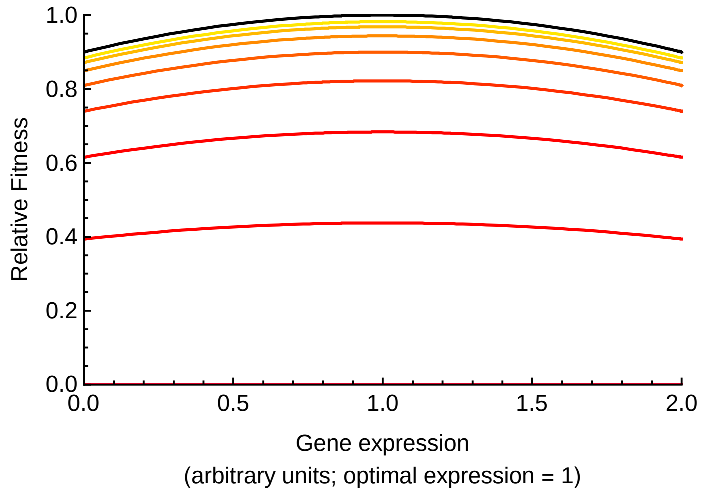

In the presence of an interaction between gene expression and antibiotic potency, we will parameterize the interaction by ‘elasticity’ or log-sensitivity parameter η ,
where an x-fold change in gene expression causes an x^{η}-fold change in antibiotic potency.
Therefore, applied antibiotic concentration ‘a’ changes to $a \times \left(\frac{g}{gwt}\right)^{\eta}$, where gwt = wildtype gene expression, g = modified level of gene expression.
We call the effective antibiotic potency a' (a-prime):

In[11]:=
```
aprime[a_, g_, gwt_, η_] := a * (g/gwt)^η
```

The Fitness-expression function accounting for an interaction between gene expression and antibiotic potency is therefore:

$$\text{Fitness} = f_g(g) \times f_a(a')$$
$$= f_g(g) \times f_a\left(a \times \left(\frac{g}{gwt}\right)^{\eta}\right)$$

In[12]:=
```
Fitness[a_, g_, gwt_, η_] := fg[g] * fa[aprime[a, g, gwt, η]]
```

In[13]:=
```
(* for simplicity here we define wildtype gene expression to be 1. This is not strictly necessary;
but it conveniently defines the modified level of gene expression, g, to be written as a fraction of the wildtype gene expression *)
GWT = 1;
```

For non-zero values of η, antibiotic treatment perturbs the optimal level of gene expression. Consequently, antibiotic concentrations greater than the wild-type Minimum Inhibitory Concentration (MIC) can be tolerated with altered gene expression.

In these plots, for the simplicity of the illustration, gene expression varies linearly over the horizontal axis scale, and therefore the mild overexpression (2x) has small effect; the next section of the file will illustrate gene expression variation over a log-scale, as has been illustrated in the manuscript, to observe the effects of larger levels of gene overexpression.

In[14]:=
```
(* η *)ETA = 1;
Show[Table[Plot[Fitness[AntibioticConcentrations⟦i⟧, g, GWT, ETA], {g, 0, 2}, PlotRange → {0, 1}, PlotStyle → plotcolors⟦i⟧],
   {i, 1, 14, 1}], AxesOrigin → {0, 0}, Frame → {{True, False}, {True, False}},
 FrameTicks → {Automatic, Automatic}, PlotRangePadding → None, BaseStyle → {FontFamily → "Arial", FontSize → 12},
 FrameStyle → Directive[Black, Thickness[Medium]], FrameLabel → {"Gene expression", "Relative Fitness"},
 PlotStyle → AbsoluteThickness[2], PlotLabel → Style["η = " <> ToString[ETA], Black, FontSize → 16]]

(* η *)ETA = 0.25;
Show[Table[Plot[Fitness[AntibioticConcentrations⟦i⟧, g, GWT, ETA], {g, 0, 2}, PlotRange → {0, 1}, PlotStyle → plotcolors⟦i⟧],
   {i, 1, 14, 1}], AxesOrigin → {0, 0}, Frame → {{True, False}, {True, False}},
 FrameTicks → {Automatic, Automatic}, PlotRangePadding → None, BaseStyle → {FontFamily → "Arial", FontSize → 12},
 FrameStyle → Directive[Black, Thickness[Medium]], FrameLabel → {"Gene expression", "Relative Fitness"},
 PlotStyle → AbsoluteThickness[2], PlotLabel → Style["η = " <> ToString[ETA], Black, FontSize → 16]]

(* η *)ETA = -0.25;
Show[Table[Plot[Fitness[AntibioticConcentrations⟦i⟧, g, GWT, ETA], {g, 0, 2}, PlotRange → {0, 1}, PlotStyle → plotcolors⟦i⟧],
   {i, 1, 14, 1}], AxesOrigin → {0, 0}, Frame → {{True, False}, {True, False}},
 FrameTicks → {Automatic, Automatic}, PlotRangePadding → None, BaseStyle → {FontFamily → "Arial", FontSize → 12},
 FrameStyle → Directive[Black, Thickness[Medium]], FrameLabel → {"Gene expression", "Relative Fitness"},
 PlotStyle → AbsoluteThickness[2], PlotLabel → Style["η = " <> ToString[ETA], Black, FontSize → 16]]

(* η *)ETA = -1;
Show[Table[Plot[Fitness[AntibioticConcentrations⟦i⟧, g, GWT, ETA], {g, 0, 2}, PlotRange → {0, 1}, PlotStyle → plotcolors⟦i⟧],
   {i, 1, 14, 1}], AxesOrigin → {0, 0}, Frame → {{True, False}, {True, False}},
 FrameTicks → {Automatic, Automatic}, PlotRangePadding → None, BaseStyle → {FontFamily → "Arial", FontSize → 12},
 FrameStyle → Directive[Black, Thickness[Medium]], FrameLabel → {"Gene expression", "Relative Fitness"},
 PlotStyle → AbsoluteThickness[2], PlotLabel → Style["η = " <> ToString[ETA], Black, FontSize → 16]]
```

Out[15]=

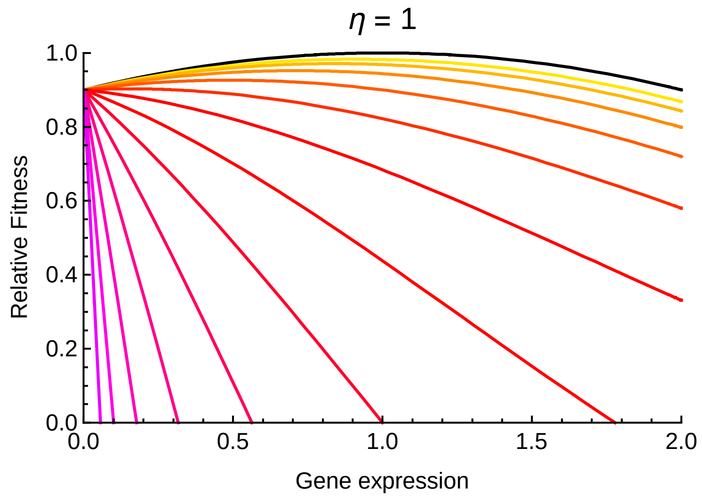

Out[17]=

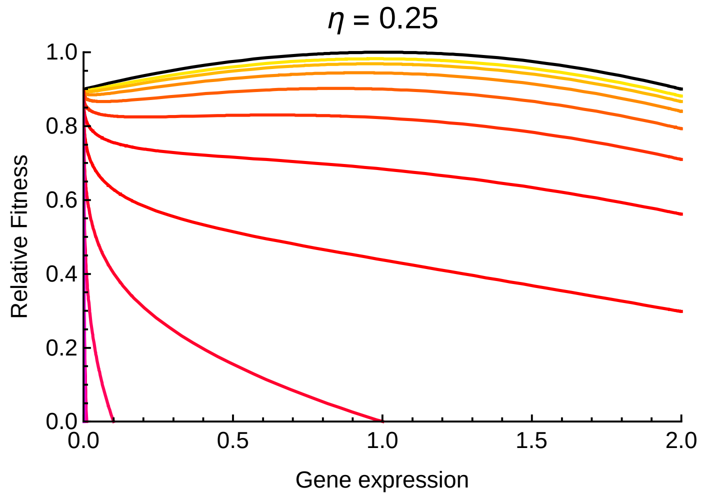

Out[19]=

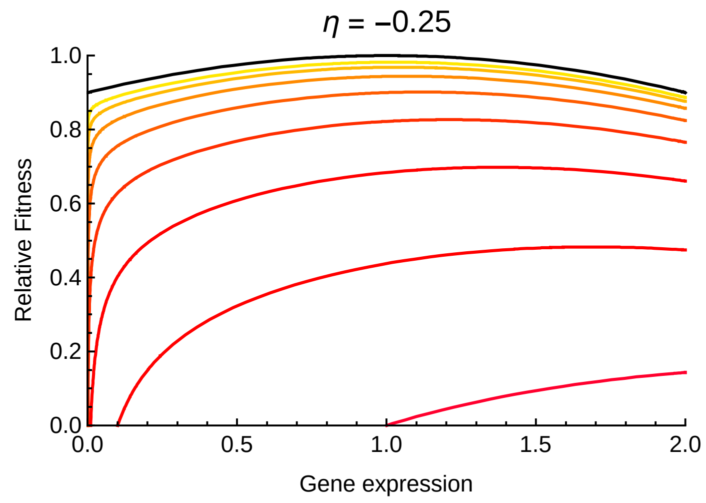

Out[21]=

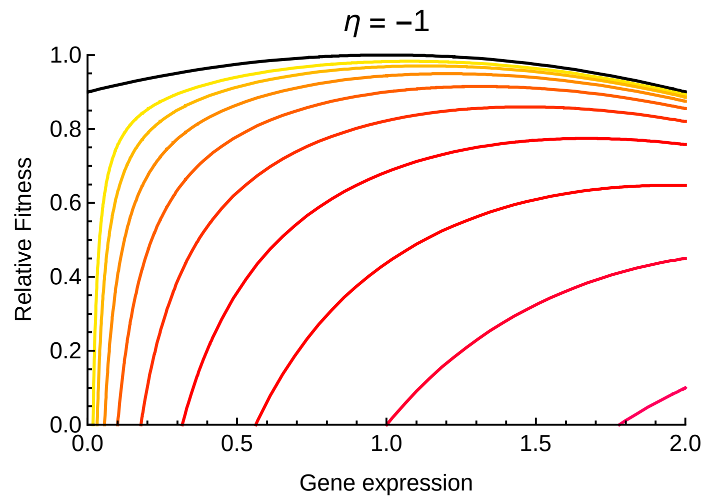

Gene expression plotted over log scale
(as plotted in the manuscripts figures, based on experiments where promoter activity was varied over an approximately 50× range)

In[22]:=

```
fg[g_] := 1 - (Log[10, g])^2 / 10
Plot[fg[10^logg], {logg, -1.5, 1.5}, PlotRange → {0, 1}, Axes → False,
 Frame → {{True, False}, {True, False}}, FrameTicks → {logframeticks, Automatic}, PlotRangePadding → None,
 BaseStyle → {FontFamily → "Arial", FontSize → 12}, FrameStyle → Directive[Black, Thickness[Medium]],
 FrameLabel → {"Gene expression\n(arbitrary units; optimal expression = 1)", "Relative Fitness"},
 PlotStyle → Directive[Black, AbsoluteThickness[2]]]
```

Out[23]=

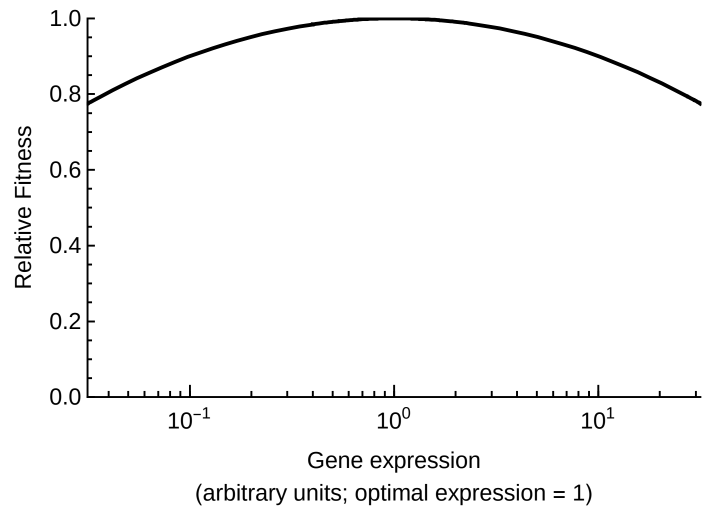

In[24]:=

```
aprime[a_, g_, gwt_, η_] := a * (g / gwt)^η
```

In[25]:=

```
Fitness[a_, g_, gwt_, η_] := fg[g] * fa[aprime[a, g, gwt, η]]
```

In[26]:=
```
(* η *) ETA = 1;
Show[Table[Plot[Fitness[AntibioticConcentrations[[i]], 10^logg, GWT, ETA], {logg, -1.5, 1.5}, PlotRange → {0, 1}, PlotStyle → plotcolors[[i]]],
  {i, 1, 14, 1}], Axes → False, Frame → {{True, False}, {True, False}},
 FrameTicks → {logframeticks, Automatic}, PlotRangePadding → None, BaseStyle → {FontFamily → "Arial", FontSize → 12},
 FrameStyle → Directive[Black, Thickness[Medium]], FrameLabel → {"Gene expression", "Relative Fitness"},
 PlotStyle → AbsoluteThickness[2], PlotLabel → Style["η = " <> ToString[ETA], Black, FontSize → 16]]

(* η *) ETA = 0.25;
Show[Table[Plot[Fitness[AntibioticConcentrations[[i]], 10^logg, GWT, ETA], {logg, -1.5, 1.5}, PlotRange → {0, 1}, PlotStyle → plotcolors[[i]]],
  {i, 1, 14, 1}], Axes → False, Frame → {{True, False}, {True, False}},
 FrameTicks → {logframeticks, Automatic}, PlotRangePadding → None, BaseStyle → {FontFamily → "Arial", FontSize → 12},
 FrameStyle → Directive[Black, Thickness[Medium]], FrameLabel → {"Gene expression", "Relative Fitness"},
 PlotStyle → AbsoluteThickness[2], PlotLabel → Style["η = " <> ToString[ETA], Black, FontSize → 16]]

(* η *) ETA = -0.25;
Show[Table[Plot[Fitness[AntibioticConcentrations[[i]], 10^logg, GWT, ETA], {logg, -1.5, 1.5}, PlotRange → {0, 1}, PlotStyle → plotcolors[[i]]],
  {i, 1, 14, 1}], Axes → False, Frame → {{True, False}, {True, False}},
 FrameTicks → {logframeticks, Automatic}, PlotRangePadding → None, BaseStyle → {FontFamily → "Arial", FontSize → 12},
 FrameStyle → Directive[Black, Thickness[Medium]], FrameLabel → {"Gene expression", "Relative Fitness"},
 PlotStyle → AbsoluteThickness[2], PlotLabel → Style["η = " <> ToString[ETA], Black, FontSize → 16]]

(* η *) ETA = -1;
Show[Table[Plot[Fitness[AntibioticConcentrations[[i]], 10^logg, GWT, ETA], {logg, -1.5, 1.5}, PlotRange → {0, 1}, PlotStyle → plotcolors[[i]]],
  {i, 1, 14, 1}], Axes → False, Frame → {{True, False}, {True, False}},
 FrameTicks → {logframeticks, Automatic}, PlotRangePadding → None, BaseStyle → {FontFamily → "Arial", FontSize → 12},
 FrameStyle → Directive[Black, Thickness[Medium]], FrameLabel → {"Gene expression", "Relative Fitness"},
 PlotStyle → AbsoluteThickness[2], PlotLabel → Style["η = " <> ToString[ETA], Black, FontSize → 16]]
```

Out[27]=

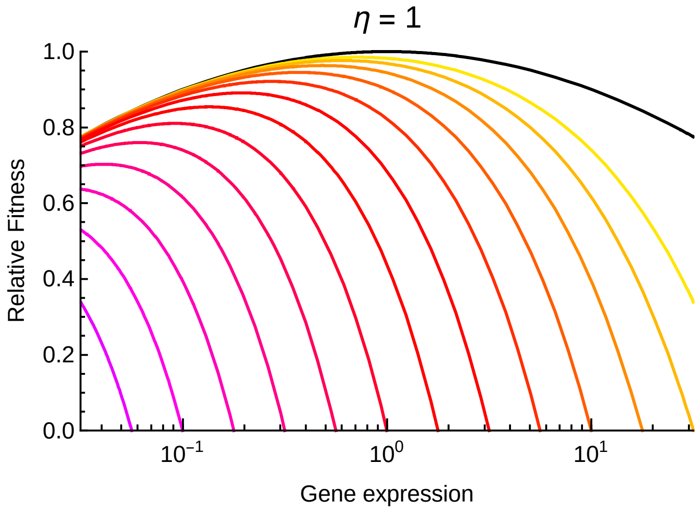

Out[29]=

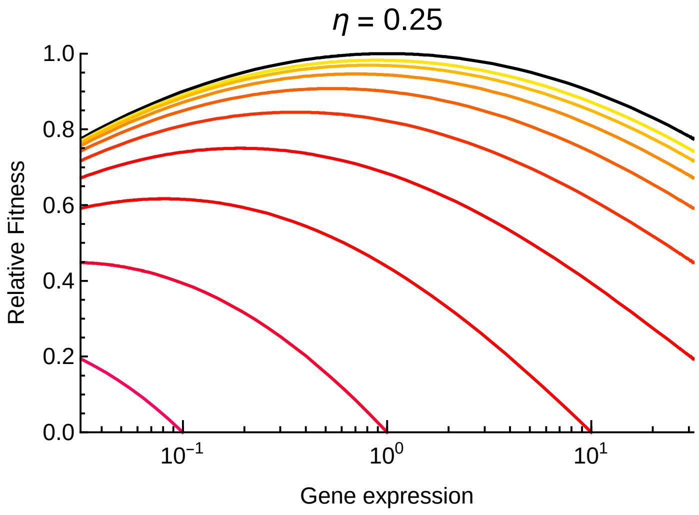

Out[31]=

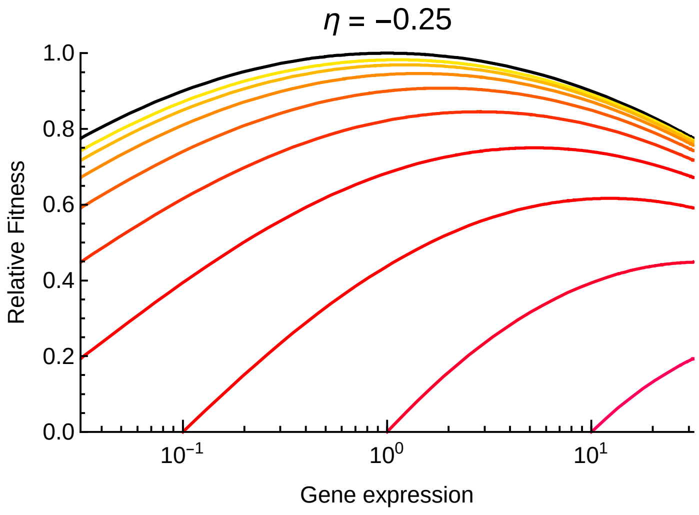

Out[33]=

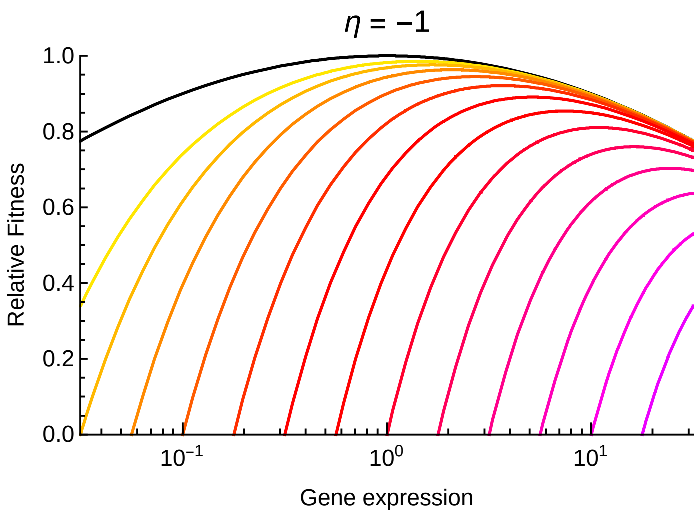
η = −1
Relative Fitness
1.0
0.8
0.6
0.4
0.2
0.0
10^−1
10^0
10^1
Gene expression
